# The functional analysis of sugar transporter proteins in sugar accumulation and pollen tube growth in pummelo (*Citrus grandis*)

**DOI:** 10.3389/fpls.2022.1106219

**Published:** 2023-01-04

**Authors:** Weiwei Xu, Ziyan Liu, Zeqi Zhao, Shuhang Zhang, Mengdi Li, Dayong Guo, Ji-Hong Liu, Chunlong Li

**Affiliations:** ^1^ Key Laboratory of Horticultural Plant Biology Ministry of Education (MOE), College of Horticulture and Forestry Science, Huazhong Agricultural University, Wuhan, China; ^2^ Hubei Hongshan Laboratory, Wuhan, China

**Keywords:** *Citrus grandis*, sugar transporter protein, gene expression, sugar accumulation, pollen tube growth

## Abstract

Sugar transporter proteins (STPs) play vital roles in sugar transport and allocation of carbon sources in plants. However, the evolutionary dynamics of this important gene family and their functions are still largely unknown in citrus, which is the largest fruit crop in the world. In this study, fourteen non−redundant CgSTP family members were identified in pummelo (*Citrus grandis*). A comprehensive analysis based on the biochemical characteristics, the chromosomal location, the exon–intron structures and the evolutionary relationships demonstrated the conservation and the divergence of CgSTPs. Moreover, CgSTP4, 11, 13, 14 were proofed to be localized in plasma membrane and have glucose transport activity in yeast. The hexose content were significantly increased with the transient overexpression of *CgSTP11* and *CgSTP14*. In addition, antisense repression of *CgSTP4* induced the shorter pollen tube length *in vitro*, implying the potential role of CgSTP4 in pummelo pollen tube growth. Taken together, this work explored a framework for understanding the physiological role of *CgSTPs* and laid a foundation for future functional studies of these members in citrus species.

## Introduction

Soluble sugars such as sucrose, glucose and fructose are the main carbohydrates from photosynthesis of most plants (Rolland et al., 2002). These sugars are the key components of carbon and energy metabolism in plants, providing the skeleton for large molecules such as proteins and nucleic acids (Smeekens and Hellmann, 2014). Besides, sugars can be used as signal transduction molecules regulating various metabolic pathways, biotic and abiotic stress responses, plant growth and development, and regulating the osmotic pressure of plant cells and solutions, thus affecting stomatal opening and closing and other activities (Rolland et al., 2002; Koch, 2004; Rolland et al., 2006; Radchuk et al., 2010; Smeekens and Hellmann, 2014; Huai et al., 2022). In plants, sugars need to pass through membrane several times on its way from the source cells to the sink organs. The cross boundary membrane uptake of sugars is a major event for nutrition in all eukaryotic cells (Tao et al., 2015). The carrier proteins mediating the transmembrane transport of sugars are called sugar transporters. Currently, the vast majority of identified sugar transporters belong to the major facilitator superfamily (MFS), which is usually composed of 12 transmembrane domains known as H^+^/sugar or Na^+^/sugar cotransporters (Yan, 2013). According to the different transport substrates, the MFS in plants is mainly divided into disaccharide transporter and monosaccharide transporters (MSTs). Among them, the main disaccharide transporter is sucrose transporters (SUTs) (Chiou and Bush, 1996), also known as sucrose carriers (SUCs), which mediate the transport of sucrose and maltose (Riesmeier et al., 1994; Kuhn and Grof, 2010). The MSTs are composed of a large gene family, which contain seven subfamilies named sugar transporters (STPs) (also known as Hexose transporters, HTs), tonoplast monosaccharide transporters (TMTs), vacuolar glucose transporters (VGTs), plastid glucose transporters (pGlcTs), early response to drought 6-like transporters (ERD6Ls), polyol/monosaccharide transporters (PMTs) and insitol transporters (INTs) (Buttner, 2007; Li et al., 2015a; Fang et al., 2020; Liu et al., 2020). In addition, there is a new class of sugar transporter, which is named as the Sugars Will Eventually be Exported Transporters (SWEETs) (Chen et al., 2010). SWEETs belong to the MtN3-Like membrane protein and have a completely different structure from the MFS sugar transporters (Gautam et al., 2022). These sugar transporters mentioned above have been reported localized in various subcellular locations such as plasma membrane, vacuolar membrane, golgi apparatus membrane, and plastid membrane (Jiu et al., 2018; Fang et al., 2020; Li et al., 2020). The diversity subcellular localization patterns confers a variety of functions for sugar transport proteins. At present, the family of sugar transporters located on the plasma membrane and vacuolar membrane are more studied.

Among the numerous sugar transporter families, the *STPs* are the most studied monosaccharides transporters, indicating the vital role of STPs in plant development or stress response. STPs are complete membrane proteins with 12 transmembrane domains and are considered to be H^+^/sugar transporters located on the plasma membrane. So far, the *STPs* have been identified and studied in many plants, including *Arabidopsis thaliana* (Buttner, 2010)*, Manihot esculenta* (Liu et al., 2018)*, Brassica oleracea* var. *capitata L.* (Zhang et al., 2019)*, Oryza sativa* (Toyofuku et al., 2000; Deng et al., 2019)*, Solanum lycopersicum* (Reuscher et al., 2014)*, Vitis vinifera* (Afoufa-Bastien et al., 2010)*, Fragaria vesca* (Jiu et al., 2018; Liu et al., 2020)*, Pyrus bretschneideri Rehd* (Li et al., 2015a)*, Malus domestica* (Wei et al., 2014)*, Dimocarpus longan Lour* (Fang et al., 2020). Based on previous reports, *STP* members are expressed in different tissues and participate in various metabolic pathways for specific physiological functions. The expression of *STPs* are also response to hormones, biotic and abiotic stresses, which further significantly affect plant development and stress resistance (Truernit et al., 1996; Buttner et al., 2000; Sade et al., 2013; Murcia et al., 2016; Murcia et al., 2018; Deng et al., 2019; Otori et al., 2019; Paulsen et al., 2019). A total of 14 AtSTPs have been reported in *A. thaliana* (Buttner, 2010), all of which are localized at the plasma membrane and are responsible for the transport of monosaccharides from the apoplastic space to the cytosol. For example, AtSTP1 is capable of transporting other hexose in addition to fructose, and is involved in the transport of monosaccharides in guard cells (Stadler et al., 2003). AtSTP2 is primarily responsible for absorbing glucose produced by callose degradation during the early stages of pollen maturation (Truernit et al., 1999). AtSTP4, AtSTP6, AtSTP8, AtSTP9, AtSTP10 and AtSTP11 are mainly responsible for the uptake of glucose into the pollen tube of *A. thaliana* and play a role in the supply of monosaccharides during the growth of the pollen tube (Rottmann et al., 2018). Heterologous expression of the apple hexose transporter gene *MdHT2.2* in tomato promotes sucrose, fructose, and glucose accumulation (Wang et al., 2020b) in response to tomato salt tolerance mechanism by balancing cytoplasmic to intercellular ion concentrations and scavenging reactive oxygen species (ROS) (Wang et al., 2020a). In wheat, *TaSTP3* is transcriptionally activated by the transcription factor TaWRKY19/61/82 during stripe rust, thereby increasing the sucrose concentration of host cells to guarantee carbon source supply for the fungus (Huai et al., 2022). In apple, MdSTP13a is found to absorb hexose and sucrose simultaneously in the process of sorbitol regulating pollen tube growth to promote the growth of apple pollen tubes (Li et al., 2020). These findings highlight the importance of STPs in plant growth, development, and stress tolerance *via* sugar transport and carbon source allocation.

The citrus is the largest fruit industry over the world. Studies have shown that the genus citrus originated from three ancestral species: *Citrus maxima*, *Citrus medica*, and *Citrus reticulate* (Wu et al., 2018). It has been suggested that domestication of citrus may have begun with the identification and asexual reproduction of selected, possibly hybrid or mixed individuals. For instance, the cultivation of pummelo (*Citrus grandis*) is cultivated from the ancestral *C. maxima* with the introgressions of the other citrus species (Xu et al., 2013; Wu et al., 2014). The fruit quality of citrus is affected by flavor substances, which mainly include soluble sugars, organic acids and volatile compounds, among which the composition and content of soluble sugars is the key factor affecting taste and thus determining fruit flavor quality (Li et al., 2012; Aslam et al., 2019). There are also some reports to support that the relationship between sugar transporters and sugar accumulation in citrus (Zheng et al., 2014). For instance, the soluble sugar-related genes in ‘Rongan’ (RA) and its mutant ‘Huapi’ (HP) kumquat were analyzed, and it was found that high sugar accumulation in HP fruit was associated with up-regulation of *SUS*, *SPS*, *TST*, *STP* and *ERD6L* genes (Wei et al., 2021). In addition, artificial thinning can increase the size and sugar content of citrus fruits, affect hormone synthesis and sugar transporter activity, and significantly improve fruit quality (Liu et al., 2022a). However, these studies have only pointed out the potential role of sugar transporters in citrus, but the characteristics and functions of sugar transporters in citrus species are still confused so far.

In this study, the genes encoding *STPs* in *C. grandis* genome were identified. The phylogenetic relationships, characteristics, structure, conserved motifs, *cis*-acting elements and collinearity of CgSTP members were analyzed, revealing the conserved and correlation between homologous and near-homologous genes. Based on the expression pattern and subcellular localization assay, CgSTP4, CgSTP11, CgSTP13 and CgSTP14 were further selected and proofed to have the hexose sugar transport activity. In addition, the function of pollen-specific expression *CgSTP4* was explored to be involved in pummelo pollen tube growth *via* antisense oligonucleotide transformation. Taken together, we identified key candidate *CgSTP* genes in sugar accumulation, which will be a great scientific significance and potential application for further investigation of the physiological functions of *CgSTPs* in pummelo or other citrus species.

## Materials and methods

### Plant materials and growth conditions

The ‘Shatian pummelo’ (*Citrus grandis*) fruits, leaves, flowers and other tissues were harvested from the Centre of Citrus Plant at Huazhong Agricultural University (Wuhan, China). The pummelo trees were maintained under standard horticultural management and prevention of plant diseases and insect pests. At the popcorn stage, flowers were picked for anther collection, and the anthers were dried in a 28°C oven. The dried anthers and released pollen were collected into 1.5 or 2.0 ml centrifuge tubes sealed with silica gel and stored in -20°C refrigerator for further use. Tobacco plants (*Nicotiana benthamiana*) were grown in the growth chamber at 23-25°C with 12h light/12 h darkness.

### Database searches and identification of STPs in *Citrus grandis*

In order to identify the *STP* genes in *C. grandis*, the whole-genome data of pummelo (Citrus grandis (L.) Osbeck.cv. ‘Wanbaiyou’ v1.0) was downloaded from the Citrus Pan-genome to Breeding Database website (http://citrus.hzau.edu.cn/index.php), which was used to obtain the gene sequences and gene annotations. The Hidden Markov Model (HMM) of the Sugar_tr domain (PF00083) from HMMER (https://www.ebi.ac.uk/Tools/hmmer/search/hmmscan) was used to search the pummelo protein database at a standard E-value < 1.0 × 10^−5^ (Finn et al., 2011; Chen et al., 2020; Mistry et al., 2021). A total of 53 hypothetical CgSTP proteins were identified. Furthermore, the conserved domain composition of 14 AtSTP protein sequences were analyzed by CD-search. Then, all the 53 putative protein sequences filtered to submit to the National Center for Biotechnology Information (NCBI, https://www.ncbi.nlm.nih.gov/Structure/bwrpsb/bwrpsb.cgi). Finally, 14 STP family members of *C. grandis* were screened out. They were named CgSTP1 to CgSTP14 based on their relationship with members of the STP family in *A. thaliana*.

### Phylogenetic tree construction and synteny correlation analysis

To explore the evolutionary relationship of *CgSTPs* between *C. grandis* and *A. thaliana*, a phylogenetic tree was constructed by maximum likelihood (ML) method using MEGAX64 software based on the protein sequences of 14 CgSTPs from pummelo and 14 AtSTPs from *A. thaliana*. The final tree is then beautified through the ITOL website. Among them, the AtSTP protein sequences were downloaded from the The Arabidopsis Information Resource website (https://www.arabidopsis.org/). In order to better understand the conservation of *STP* genes in evolution, the collinear correlation analysis between species was carried through the MCScanX (Wang et al., 2012) of TBtools software by offering gene annotation and the whole genome sequence of *A. thaliana* and *C. grandis*.

### Amino acid characteristic and gene structure prediction

The physicochemical properties of the proteins, which included amino acid number (AA), molecular weight (MW), theoretical isoelectric point (PI), and grand average of hydropathy (GRAVY), were obtained in the ExpasyProtParam server (https://web.expasy.org/compute_pi/) (Wilkins et al., 1999). The number of transmembrane domains was predicted by the NovoPro url (https://www.novopro.cn/tools/tmhmm.html). The WoLF PSORT server (https://wolfpsort.hgc.jp/) was applied to predict protein subcellular localization. The gene structure annotation and CDS files of *C. grandis* were downloaded from the Citrus Pan-genome to Breeding Database website, and the *STP* genes information was extracted by TBtools using accession number, followed by the Gene Structure Display Server 2.0(http://gsds.gao-lab.org/)to visualize the exon–intron structure of these genes (Hu et al., 2015).

### Protein motif and *cis*-acting elements analysis

To further explore the gene structure of *CgSTPs*, the MEME Suite web server (https://meme-suite.org/meme/tools/meme) (Bailey and Elkan, 1994; Bailey et al., 2009) was used to predict their protein sequences with a maximum number of motif groups of 12 for conserved motifs, any number of repeats, and an optimal width of the motif ranging from 15 to 60 amino acids. The promoter sequences (2 kb of genomic DNA sequence upstream of the translation initiation codon) of the *CgSTP* genes were obtained from *C. grandis* genome files and submitted to the PlantCARE database (http://bioinformatics.psb.ugent.be/webtools/plantcare/html/) to predict *cis*-elements in the promoter (Rombauts et al., 1999; Lescot et al., 2002).

### RNA extraction, cDNA synthesis and quantitative real-time analysis

Each 0.2g pummelo tissues, including leaves, flowers and juice sac were snap frozen and ground into fine powder in liquid nitrogen with three independent repetitions. RNA from common tissues was extracted using the Ominplant RNA Kit (Cwbio. Jiangsu, China). And for polyphenol polysaccharide containing tissues, RNA was extracted using the RNAprep Pure Plant Plus Kit (TIANGEN, Wuhan, China) according to the instructions. The concentration and quality of the extracted RNA were confirmed *via* spectrophotometer and agar gel electrophoresis. Then the EasyScript One-Step gDNA Removal and cDNA Synthesis SuperMix (TransGen Biotech, Beijing, China) reverse transcribed RNA into cDNA. Finally, three technical replicates of qRT-PCR were performed using SYBR Green Supermix kit according to the manual *via* the Applied biosystem^®^QuantStudio™ 7 Flex Real-Time PCR System (ABI, Los Angeles, CA, USA), and 2*^-ΔΔCT^* method (Udvardi et al., 2008) was used to calculate and analyze the obtained data.

### Subcellular localization of CgSTPs

The CDSs of the *CgSTP* genes were amplified using gene-specific primers without stop codons (**Supplementary Table 1**). The homologous recombination approach (Rozwadowski et al., 2008) was used to concatenate these target genes with the YFP101 vector, which contained a C-terminal yellow fluorescent protein (YFP) driven by 35S promoter. After the correct comparison between the sequencing results of Tsingke Biological Company (Wuhan, China) and the CDS of the genome through DNAMAN software, the recombinant plasmid was transferred into *Agrobacterium tumefaciens* GV3101 (Krenek et al., 2015) (Weidi, Wuhan, China). Then, *A. tumefaciens* containing the recombinant plasmid was injected into tobacco leaves for transient expression (Kato et al., 2002). Finally, the scanning confocal microscope (Leica TCS-SP8, Wetzlar, Germany) was used to image YFP fluorescence with an excitation wavelength of 514 nm and emission at 520-551 nm.

### Functional characterization of *CgSTPs* by heterologous expression in yeast

The CDSs of the target gene were cloned with gene-specific primers containing stop codons (**Supplementary Table 1**). The yeast expression vector pDR196 was ligated with the gene of interest by homologous recombination, followed by transformation of the recombinant plasmids into the hexose transport deficient yeast strain *EBY.VW4000* by lithium acetate method (Soni et al., 1993). The empty vector pDR196 was used as negative control, and the recombinant vector pDR196-*AtSTP13* was served as a positive control (Riesmeier et al., 1992; Rottmann et al., 2016; Li et al., 2020). The transformed cells in *EBY.VW4000* were pre-incubated in liquid Synthesis Defect (SD)-Ura medium supplemented with 2% maltose (w/v) as the sole carbon source until the OD_600_ value reached 0.6-0.8 (Wieczorke et al., 1999). Four serial dilutions (10×) of yeast cells were then plated on solid SD-Ura medium containing 2% maltose or glucose as the sole carbon source. The samples were cultured at 30°C for 3 days and then observed and photographed.

### Transient expression in tobacco leaves and sugars content analysis

The CDS(s) of *CgSTP* genes were cloned with gene-specific primers without termination codons (**Supplementary Table 1**) and inserted into the overexpression vector pK7WG2D with the 35S promoter (Pi et al., 2022). After that, the final recombinant plasmid was transformed into *Agrobacterium tumefaciens* GV3101, which was further used for injection of tobacco leaves for transient overexpression. The control with empty vector and the recombinant plasmid carrying the target genes (*CgSTP(s)*-2D) were transformed into the same leaf, as control in the left and *CgSTP(s)*-2D in the right part of the leaf. Samples were taken on the third day after transformation. The relative gene expression level was determined by RT-PCR assay (the primers were listed in **Supplementary Table 1**). Then, the content of monosaccharides was measured by GC method as described previously (Liu et al., 2022b). Soluble sugars were extracted in 75% methanol with ribitol (0.12 mg per sample) added as an internal standard and then derivatized sequentially with methoxyamine hydrochloride and N-methyl-N-(trimethylsilyl) trifluoroacetamide (MSTFA). After derivatization, the metabolites were analyzed using a GC equipment (Fuli GC-9720 Pluse, Zhejiang, China) with a HP-FFAP column (30.00 m * 0.32 mm * 0.25 μm) and a 5 m Duraguard column (Agilent Technologies, Palo Alto, CA, USA). Sugar content was quantified based on standard curves generated for each sugar (glucose and fructose) and an internal standard. All results were determined at least three biological replicates.

### *In vitro* pollen tube growth experiments and antisense oligonucleotide transfection

The pollen grains were cultured in liquid germination media (0.02%[w/v]MgSO_4_, 0.01%[w/v]KNO_3_, 0.03%[w/v]Ca(NO_3_)_2_, 0.01%[w/v]H_3_B0_3_, 15%[w/v]PEG-4000, 10%[w/v]Sucrose, and liquid NaOH to pH 6.0) (Liang et al., 2017) for pollen tube length assay. Only 1 μl of transfection agent (Biosharp, China) and 1 μl of transfection agent plus 1 μl of oligonucleotide primers (ODN concentration of 100 μM) were added as the control group, and 1 μl of transfection agent plus 1 μl of antisense oligonucleotide primers were added as the experimental group (Meng et al., 2014). Three independent experiments were performed for each treatment, and the same amount of pollen grains were added for germination culture at 25°C in a constant temperature and dark environment. At the 4 h, 6 h and 8 h of culture, the germinated pollen grains were observed and photographed by a type microscope, and then each biological replicate at least 100 germinated pollen grains were measured *via* image software. **Supplementary Table 1** lists the oligonucleotide primer sequences used in this study. For gene expression assay, individual duplicate pollen tubes were collected into a 10 ml centrifuge tube and centrifuged under 12000g at 4°C for 10 min. The samples could be re-suspended once in pure water and centrifuged again to remove the supernatant under the same conditions, after that the samples were snap frozen in liquid nitrogen for RNA extraction and gene expression assay by qRT-PCR.

## Result

### Genome-wide identification and basic information analysis of *STP* family genes in *C. grandis*

**Figure 1:**
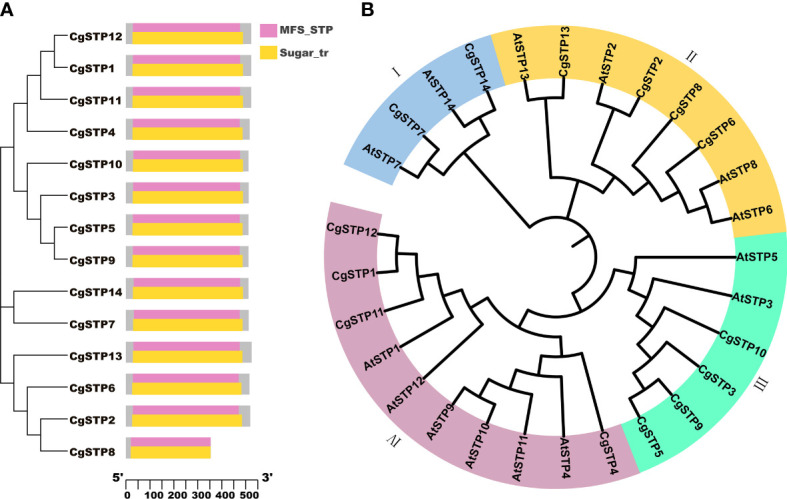
Phylogenetic analysis of STP members from *C grandis* and *A thaliana*, and conserved domain analysis of CgSTPs. **(A)** The conserved domain analysis was performed through the NCBI Batch CD-search tool. **(B)** A total of 14 STP protein sequences from *C grandis* and 14 STP protein sequences from *A thaliana* were aligned by ClustalW method. The MEGAX program was applied to construct the phylogenetic tree by the ML method in the default parameters. The beautification of the tree was carried out on the iTOL website.

Using the Hidden Markov Model (HMM) and the conserved domains of MSF_STP (cd17361) and Sugar_tr (pfam00083) in *STPs*, a total of 14 *STP* family members were screened in the *C. grandis* genome database *via* the HMMER and Batch CD-Search websites (**Table 1**; **Figure 1** and **Supplementary File 1**). Following the distant genetic relationship with *Arabidopsis STP* family genes, the 14 pummelo *CgSTPs* were named as *CgSTP1* - *CgSTP14* (**Table 1**). Furthermore, the phylogenetic tree was constructed based on the protein sequences encoded by *CgSTP* genes in *C. grandis* and *AtSTP* genes in *A. thaliana via* MEGAX64 (**Figure 1B**). The results showed that the STP proteins could be classified into four groups (**Figure 1B**). Among them, tow CgSTPs (CgSTP7 and CgSTP14) together with two AtSTPs from *Arabidopsis* were attributed to Group I. Group II consisted of four CgSTPs (CgSTP2, CgSTP6, CgSTP8 and CgSTP13), and four AtSTPs. Four CgSTPs (CgSTP3, CgSTP5, CgSTP9 and CgSTP10) clustered with two AtSTPs in Group III. Group IV contained four CgSTPs (CgSTP1, CgSTP4, CgSTP11 and CgSTP12) and six AtSTPs. Furthermore, a total of two sister pairs of CgSTPs were observed in the phylogenetic tree, including CgSTP5 - CgSTP9 and CgSTP1- CgSTP12 (**Figure 1B**). The result indicated that the *STP* family members are closely related, which is also a good reference for renaming. Moreover, the position and evolutionary relationships of *CgSTP* members at the chromosomal level were visualized by TBtools based on the gene structure annotation file (**Supplemental Figure 1**). Interspecific co-linearity analysis of *STP* genes revealed that eight pairs of genes are orthologous in two species between *C. grandis* and *A. thaliana*, indicating that the STP family was widespread in higher plants and strongly conserved during the evolutionary process.

**Table 1:** The information of *CgSTP* genes.

Gene name	Accession number ^1^	Chr ^2^	AA ^3^	Mw (Da) ^4^	pI ^5^	GRAVY ^6^	TMD ^7^	Prediction Location(s) ^8^
CgSTPl	Cglg019290.l	1	522	57205.98	9.16	0.458	12	plas: 9, vacu: 3
CgSTP2	Cg8g023770. l	8	518	57190.81	9.15	0.509	11	plas: 9, vacu: 3
CgSTP3	Cg9g023330. l	9	512	55444.33	9.51	0.537	10	vacu: 8, plas: 4
CgSTP4	Cg7g013990.l	7	516	56327.43	8.89	0.596	12	plas: 6, vacu: 4
CgSTP5	Cg9g023340. l	9	511	55553.25	9.29	0.525	12	vacu: 9, plas: 4
CgSTP6	Cg9g005200. l	9	515	56567.06	8.9	0.687	12	plas: 7, vacu: 5
CgSTP7	Cg4g024730.2	4	512	55864.82	9.38	0.484	12	plas: 9, vacu: 3
CgSTP8	Cg9g0052 l 0.1	9	353	38734.75	9.33	0.567	8	vacu: 6, plas: 5
CgSTP9	Cg9g023350.2	9	511	55543.56	9.56	0.527	12	plas: 8, vacu: 3
CgSTPl0	Cg9g023370. l	9	511	55472.2	9.52	0.496	10	vacu: 10, plas: 3
CgSTPll	Cg2g041230. l	2	522	57887.94	8.86	0.472	12	plas: 6, vacu: 4
CgSTP12	Cg6g019080. l	6	522	57502.33	8.94	0.493	12	plas: 10, vacu: 2
CgSTP13	Cg9g005230. l	9	524	57802.91	9.11	0.469	12	plas: 11, vacu: 2
CgSTP14	Cglg012330. l	1	511	56065.11	9.03	0.543	12	plas: 9, vacu: 3

^1^ Accession number consisted of the CPBD

^2^ Chromosome location.

^3^ Number of amino acids of the deduced amino acid sequence.

^4^ Mw(Da) was the molecular weight.

^5^ The pl was the theoretical isoelectric point.

^6^ GRAVY was the grand average of hydropathicity.

^7^ TMD was the number of transmembrane domains, as predicted by the NovoPro.

^8^ The prediction of protein localization in cells via WoLF PSORT 11; Plas, plasma membrane; Vacu, vacuolar membrane.

According to the genomic information of *C. grandis* chromosomes, the distribution of 14 *STP* genes on the chromosome was analyzed. It was found that all *CgSTP* members were unevenly distributed on seven chromosomes, with the most abundantly distributed 7 genes on chromosome 9 (**Table 1** and **Supplemental Figure 1**). Protein sequence characteristics, including number of amino acids (AA), molecular weight (Mw), theoretical isoelectric point, grand average of hydropathicity (GRAVY), transmembrane domain (TMD) and subcellular localization prediction, were also analyzed. As shown in **Table 1**, the average STP protein length was 504 aa, with the longest 524 aa from CgSTP13 and the shortest 353 aa from CgSTP8. Accordingly, the molecular weight of these proteins ranged from 38.7 kDa (CgSTP8) to 57.9 kDa (CgSTP11). The theoretical isoelectric points ranged from 8.86 (CgSTP11) to 9.56 (CgSTP9), which were all weakly basic. According to the relevant principle of grand average of hydropathicity (GRAVY), CgSTP1, CgSTP7, CgSTP10, CgSTP11, CgSTP12 and CgSTP13 were defined as amphoteric proteins with the value between -0.5 to 0.5, while the other proteins were hydrophobic proteins with more positive value (**Table 1**). More importantly, the number of CgSTPs transmembrane domains was presented from 8 to 12 based on the prediction by the NovoPro website. The vast majority of CgSTP members had 12 transmembrane domains, which was consistent with the characteristics of the MFS superfamily (Yan, 2013). In addition, the subcellular localization prediction of the protein sequences of *CgSTP* genes was performed *via* the WoLF PSORT (**Table 1**). Most of the CgSTP proteins are predicted to be plasma membrane proteins, but some are vacuolar membrane proteins, including CgSTP3, CgSTP5, CgSTP8 and CgSTP10, which are different from the localization of STP proteins in other species. These results indicate that CgSTP proteins may play different roles in sugar transport relying on their protein characterization and subcellular localization.

### Gene structure, conserved motifs and promoter *cis*-acting elements analysis of the *CgSTPs*

**Figure 2:**
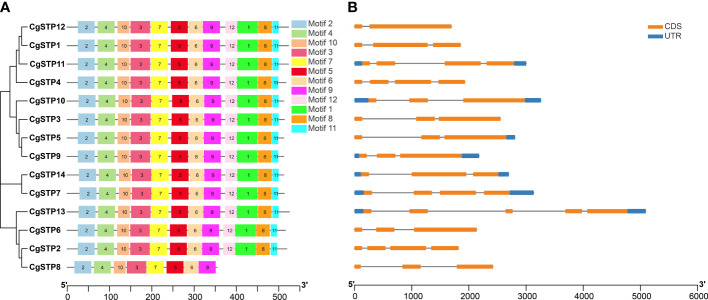
Motif, gene structure and phylogenetic relationship analysis of *CgSTP* genes. **(A)** The motif analysis was performed on the MEME. Twelve motifs were identified. The detail of motif sequence information was presented in **Supplemental Table 2**. **(B)** The gene exon–intron structure analysis. The blue color was the untranslated region, while the orange was the coding sequence. The line without color referred to introns. The neighbor-joining phylogenetic tree of the *CgSTP* genes sequences was constructed using 1000 bootstrap replicates by MEGAX64.

To understand the structure of *CgSTP* genes, exons, introns, and untranslated regions were analyzed *via* GSDS2.0 and TBtools. It was found that *CgSTP1*, *CgSTP2*, *CgSTP3*, *CgSTP4*, *CgSTP6*, *CgSTP8*, and *CgSTP12* didn’t have upstream and downstream UTRs. While *CgSTP5* didn’t have upstream UTR, and the other six members all contained upstream and downstream UTRs (**Figure 2B**). Besides, all members contained exons and introns, but differed in specific numbers and locations. The exon number of the *CgSTP* genes ranged from 2 to 5 (**Figure 2B**). *CgSTP13* gene had five exons, whereas *CgSTP12* only had two exons. The different of gene structures might contribute to the functional diversity of closely related *STP* genes. Moreover, 12 putative conserved motifs were predicted through MEME analysis in most CgSTP proteins, and all these motifs were arranged in a stable order in the protein sequence (motif 2, motif 4, motif 10, motif 3, motif 7, motif 5, motif 6, motif 9, motif 12, motif 1, motif 8, and motif 11) (**Figure 2A**). The length of the conserved motifs ranges from 16 to 50 amino acids, and these 12 motifs are contained in all CgSTP proteins, except for CgSTP8 only has 8 motifs (**Figure 2A**; **Supplementary Table 2**). The high uniformity of these conserved motifs fully reflected the relatively conserved function of CgSTP proteins in the evolutionary process.

*Cis*-elements are important molecular switches involved in the regulation of gene transcription during plant growth and development or abiotic stress response. To detect regulatory factors and predict *cis*-elements of *CgSTPs*, each of the 2 kb promoter region (upstream of the start codon of the gene) of 13 *CgSTP* genes was retrieved from the *C. grandis* genome sequence. But for the *CgSTP2* gene, the identified sequence was shorter than 2 kb (1047bp), since the presence of another gene located less than 2 kb upstream of *CgSTP2* start codon site (**Supplementary File 2**). Finally, 14 *cis*-elements in the promoter region of *CgSTP* genes were predicted by PlantCARE database (**Supplementary Figure 2A**). A heat map was further constructed to show the frequency of different *cis*-elements (**Supplementary Figure 2B**). These predicted *cis*-elements respond to different phytohormones (Gibberellin, Abscisic acid, Auxin, Methyl jasmonate, Salicylic acid) and various environmental factors, as well as abiotic and biotic stresses. Among them, hormone-related elements were widely presented in the promoter region of *CgSTPs*, so it was suspected that hormones play a potential role in regulating the expression of *CgSTP* genes. Besides, the anaerobic induction regulator is presented in the promoters of all genes except *CgSTP2*, which suggested that these genes might be induced by anaerobic conditions, such as waterlogging stress. In addition, some the other gene-specific *cis*-elements were also identified in the promoter region of the *CgSTP* genes, like the MYB binding site involved in drought-inducibility, low-temperature responsiveness, and the *cis*-acting element involved in defense and stress responsiveness, which provided the possibility to regulate gene expression and respond to various conditions of *CgSTPS*. Overall, *cis*-acting element analysis will provide a good reference for further studies on transcriptional regulation of *CgSTP* members.

### Expression profiles of *CgSTP* Genes in different tissues

**Figure 3:**
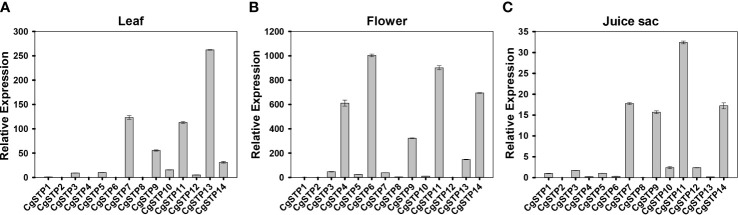
Relative expression patterns of *CgSTPs* in different tissues. **(A)** Leaf. **(B)** Flower. **(C)** Juice sac. The relative expression of all *CgSTP* members was determined by qRT-PCR assay. The *CgActin* was defined as the reference and the *CgSTP1*’s expression level was normalized as 1. Data are mean ± SE (n=3).

The spatiotemporal expression is a critical aspect in determining gene function. Accordingly, three different tissues of leaf, flower and juice sac of ‘Shatian pummelo’ were collected to perform the tissue expression pattern analysis of *STP* family members *via* qRT-PCR assay (**Figure 3**). The results showed that *CgSTPs* were widely expressed in leaf, flower and juice sac. Among them, *CgSTP7*, *CgSTP9*, *CgSTP11*, *CgSTP13* and *CgSTP14* had a higher expression level in two or three different tissues (**Figure 3**). For instance, *CgSTP13* was highly expressed in leaf and flower tissues (**Figures 3A, B**), while *CgSTP7* was highly expressed in leaf and juice sac tissues (**Figures 3A, C**). *CgSTP9*, *CgSTP11* and *CgSTP14* were highly expressed in leaf, flower and juice sac (**Figure 3**), implying that these members should have a universal functions in different tissues. What’s more, the tissue-limited expression pattern was also presented that *CgSTP4* and *CgSTP6* were specifically expressed in flower (**Figure 3B**). For some other members were expressed at relatively low levels in all three tissue, such as *CgSTP1*, *CgSTP2*, *CgSTP8* and *CgSTP10*, which might be expressed at specific developmental stage or in response to certain stimuli such as abiotic and biotic stresses. Taken together, these expression patterns in different tissues suggested that *CgSTPs* should have distinct physiology functions throughout the plant.

### Functional characterization of CgSTPs by heterologous expression in yeast

**Figure 4:**
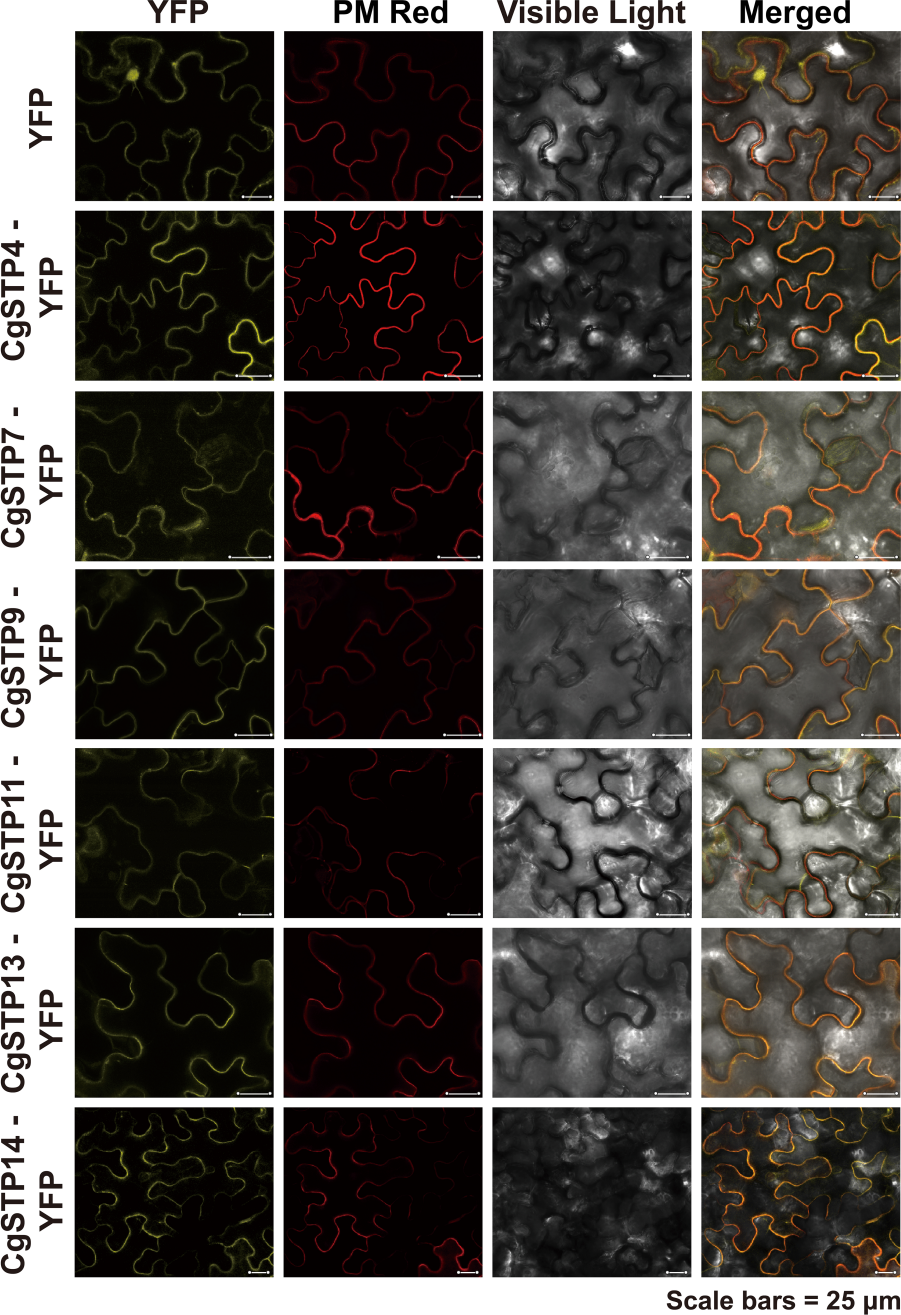
Subcellular Localization of CgSTPs. Tobacco leaves expressing 35S:YFP or 35S:CgSTP(s)-YFP, in colocalization with plasma membrane Deep Red marker, were visualized with a confocal laser microscope. Scale bars = 25 μm.

**Figure 5:**
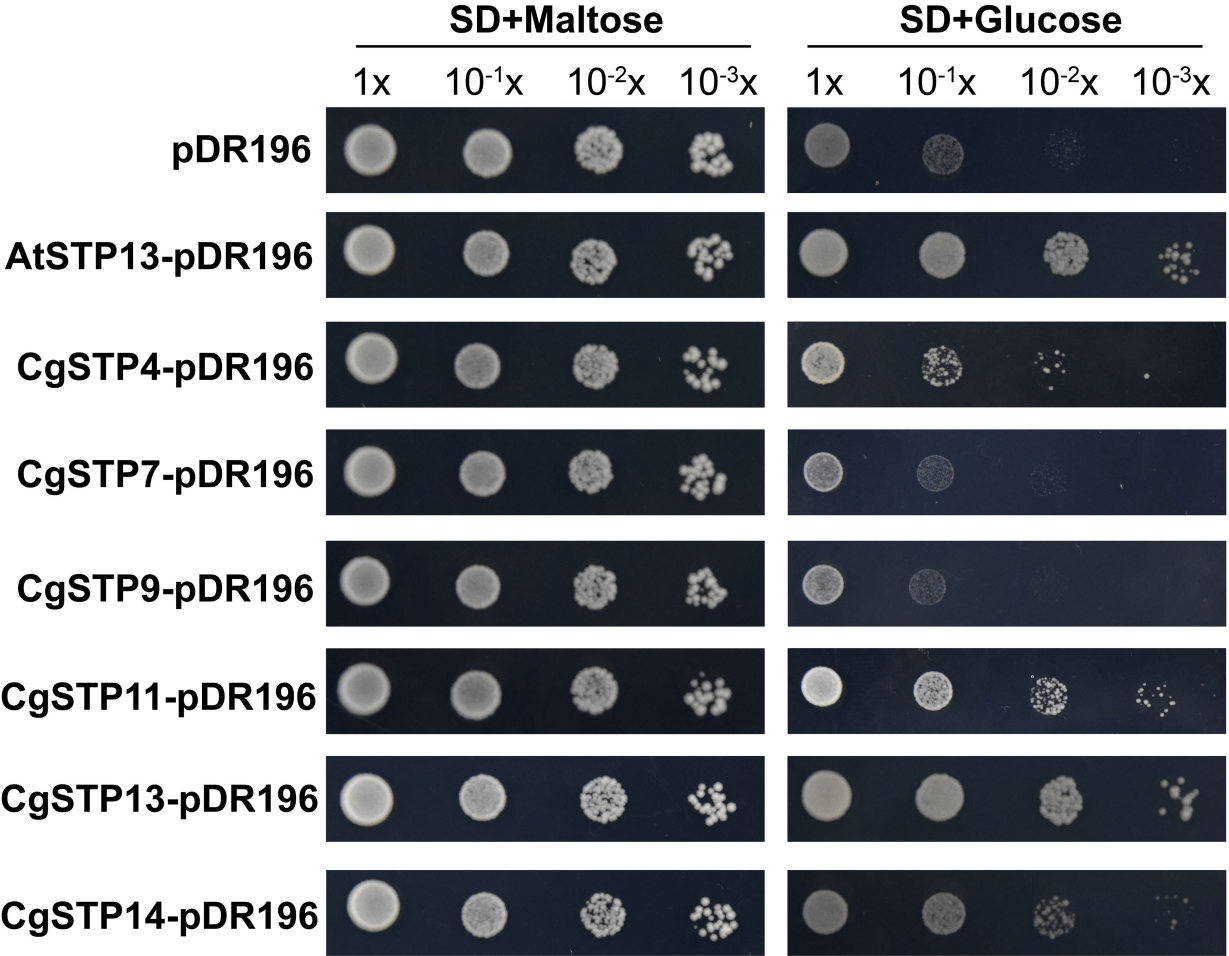
The sugar uptake assay of *CgSTPs* in Yeast (*S. cerevisiae*) Strain *EBY.VW4000*. Yeast growth assay of *EBY.VW4000* transformed with pDR196 empty vector, *AtSTP13*-pDR196 or *CgSTPs*-pDR196 alone on 2% (w/v) maltose or glucose SD-Ura medium.

To investigate the transport function of *CgSTPs*, the subcellular localization was first identified. According to the expression level, the 6 higher expressed genes (*CgSTP4*, *CgSTP7*, *CgSTP9*, *CgSTP11*, *CgSTP13*, *CgSTP14*) were selected for further analysis. The cell membrane subcellular localization of CgSTPs were observed by co-expressing the YFP fusion proteins and the plasma membrane marker (PM marker) in tobacco leaves (**Figure 4**), which is consistent with the prediction results of the WoLF PSORT (**Table 1**). Based on previous reports, STPs had hexose sugar (like glucose and fructose) transport activity (Buttner et al., 2000; McCurdy et al., 2010; Liu et al., 2018; Kong et al., 2022). To characterize the transport properties of the CgSTP proteins, the monosaccharide uptake incompetent yeast mutant strain *EBY.VW4000* (only grow on maltose medium) was applied for sugar uptake assay (Wieczorke et al., 1999). For that, the yeast expression vector pDR196 containing CgSTP4, CgSTP7, CgSTP9, CgSTP11, CgSTP13, CgSTP14 were respectively transformed into the *EBY.VW4000*, and the empty vector and AtSTP13 were used as negative and hexose-uptake positive control in yeast growth assay. Glucose was added as sole carbon source to detect the sugar transport activity of CgSTPs. All yeast cells grew well on the synthesis deficient (SD-Ura) medium containing 2% maltose (**Figure 5**), indicating the successfully expression of vector in yeast. As expected, negative control yeast cells transformed with empty vector pDR196 did not grow on glucose medium, while positive control yeast cells transformed with CgSTP13-pDR196 grew normally on glucose medium (**Figure 5**). For yeast cells carrying CgSTP4, CgSTP11, CgSTP13, and CgSTP14 could be grown on SD medium containing glucose, suggesting that these four CgSTP proteins could have glucose transport activity. In addition, the other two CgSTP members (*CgSTP7* and *CgSTP9*) were unable to recovery yeast growth on glucose medium (**Figure 5**), implying that these two *CgSTP* members didn’t have or had a weak hexose transport activity. The subcellular localization and the verification of the monosaccharide transport activity of *CgSTPs* would provide a foundation for further physiological function research.

### Validation of sugar accumulation by transient overexpression of *CgSTPs*

**Figure 6:**
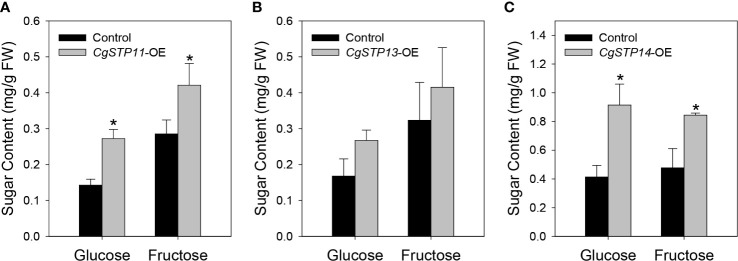
Determination of monosaccharide content in *CgSTPs* transient overexpression samples. The content of glucose and fructose in *CgSTP11* **(A)**, *CgSTP13* **(B)**, *CgSTP14* **(C)** transient overexpression tobacco leaves. Data are mean ± SE (n=3).

To verify and analyze the sugar accumulation function of *CgSTPs*, the genes that have been confirmed to have transport activity and highly expression level in fruit or leaf tissues were selected. The overexpression vectors of pK7WG2D-*CgSTP11*, *CgSTP13*, *CgSTP14* were constructed under the 35S promoter. After that, the final expression vectors containing *CgSTP11/13/14*, and the empty vector control were transformed into tobacco leaves for transient overexpression (**Supplementary Figure 3**). In consideration of the hexose transport activity, the main monosaccharides’ (glucose and fructose) content were determined by GC-FID method (Li et al., 2015b). As shown in **Figure 6**, the glucose and fructose contents with the overexpression of target *CgSTPs* were increased in comparison with the control. For *CgSTP11* and *CgSTP14*-OE samples, both glucose and fructose had a significantly higher level, implying that they did have the function of accumulating monosaccharides in plant. Overall, the transient overexpression assay provide the important clues for further more detail research of CgSTPs in citrus species.

### Antisense repression of *CgSTP4* reduces pollen tube growth

**Figure 7:**
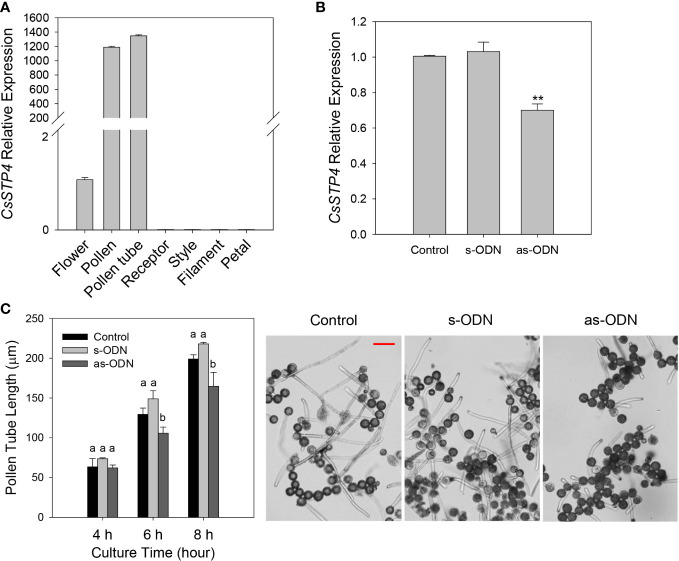
The pollen tube growth was inhibited with the lower expression of *CgSTP4*. **(A)** The expression of *CgSTP4* in different tissues of flower was determined by qRT-PCR. The *CgActin* was defined as the reference and the *CgSTP4*’s expression level in flower tissue was normalized as 1. **(B)** Expression levels of *CgSTP4* in pollen tubes transfected with antisense or sense oligonucleotide of *CgSTP4* (s-CgSTP4, as-CgSTP4), or transfection agent alone (Control). The *CgActin* was defined as the reference and the *CgSTP4*’s expression level in control condition was normalized as 1. Data are mean ± SE (n=3). * Represents significant difference in comparison with control using Student’s t test at *p* < 0.05. **(C)** Pollen tube growth assay after transfection with as-CgSTP4, s-CgSTP4, or transfection agent alone (Control). Scale bar = 50 μm. Data are mean ± SE (n=3). Letters **(A–B)** indicate significant differences at each sampling point (*p* < 0.05) using Duncan’s multiple range test (MRT) after ANOVA.

According to the previous report, sugar transport proteins were essential for pollen tube fast growth (Cheng et al., 2015; Rottmann et al., 2018; Li et al., 2020). In this study, it was found that *CgSTP4* had the specifically high expression level in flower (**Figure 3**). Therefore, the expression profile of *CgSTP4* in flower and various tissues of flower, including pollen, pollen tube, receptor, style, filament and petal of *C. grandis*, were further detected by qRT-PCR. The results revealed that *CgSTP4* was highly expressed in pollen grains and pollen tubes (**Figure 7A**). Given the importance of sugar absorption for pollen tube growth, we explored the potential role of *CgSTP4* in pollen tubes *via* the antisense oligonucleotide transfection assay (Meng et al., 2014; Meng et al., 2018). Compared with control and sense oligonucleotide transfection, the expression of *CgSTP4* can be significantly inhibited by as-ODN treatment during the germination of pollen in ‘Shatian pummelo’ (**Figure 7B**). As expected, the pollen tube length was shorter with the lower expression of *CgSTP4* (**Figure 7C**), indicating that *CgSTP4* plays an important potential role in the sugar uptake for the growth of pollen tubes in *C. grandis*.

## Discussion

Sugar transport proteins (STPs or HTs) are mainly involved in the absorption and transport of hexose in plants. They have been reported to play a key role in plant response to biotic or abiotic stresses, growth and development (Fotopoulos et al., 2003; Buttner, 2010; Slewinski, 2011). Moreover, the sugar accumulation in plant, especially in fruit tissues, is closely related to STPs. So far, there has been no systematic study on the STPs, including their expression profile, localization, physiological functions in citrus, which is the most productive fruit in the world. The acquisition of genome sequence of citrus species provides a good opportunity to identify STP family members. Here, a total of 14 CgSTPs were identified by BLAST search and HMMER analysis of *C. grandis* genome. All selected STP proteins contained MSF_STP (cd17361) and Sugar_tr (pfam00083) conserved domains (**Figure 1**), which belong to the STP family of the MFS (Yan, 2013). The amino acid number of CgSTPs is between 353 aa (*CgSTP8*) and 524 aa (*CgSTP13*) (**Table 1**), which is similar to the other reported STP families in *Arabidopsis* (Buttner, 2010), tomato (Reuscher et al., 2014), strawberry (Jiu et al., 2018; Liu et al., 2020) and pear (Li et al., 2015a), indicating the relatively stable of STPs during the whole evolutionary process. Besides, this study was the first to comprehensively analyze the gene structure, conserved motifs and *cis*-acting elements of *CgSTP* family. The expression pattern and function analysis of *CgSTP* gene were further studied.

Phylogenetic analysis presented that CgSTP proteins were classified into four groups in combination with AtSTPs in each group (**Figure 1**). The result of collinear correlation revealed eight pairs of homologous genes between pummelo and *Arabidopsis* (**Figure S1**), indicating potential similarities in evolutionary relationship and functions between the two plants. This will provide a useful reference for the future functional research of CgSTPs. It has been reported that most STP proteins have 12 transmembrane domains (TMD1-TMD12), including N-domain (TMD1-TMD6) and C-domain (TMD7-TMD12) (Hirai et al., 2002; Yan, 2013). In this study, 10 of the 14 CgSTP proteins contained the all of 12 TMDs, while CgSTP2 had 11 TMDs and 2 CgSTP had 10 TMDs (**Table 1**). However, CgSTP8 carries only 8 TMDs (**Table 1**), alone with a missing sequence in the middle of the protein. These results suggested that loss of N-terminal or C-terminal regions may have occurred in some CgSTP members during evolution. Consistent with this, similar STP protein structures have been observed in cassava (Liu et al., 2018), tomato (Reuscher et al., 2014), and grapevine (Afoufa-Bastien et al., 2010). As the transmembrane transports, most of the CgSTP were predicted to be localized in the plasma membrane, while a few be localized the vacuolar membrane based on the WoLF PSORT database (**Table 1**). Subcellular localization assay indicated that CgSTP4, CgSTP7, CgSTP9, CgSTP11, CgSTP13 and CgSTP14 were located to the plasma membrane (**Figure 4**), which was consistent with the predicted results. Accordingly, the most plasma membrane-localized members were proofed to have hexose sugar transport activity *via* the yeast mutant recovery growth assay (**Figure 5**).

In addition, the conserved motif analysis was carried out to further reveal the relationship between the various members and the potential roles of CgSTPs. Interestingly, the result demonstrated that CgSTPs contained all motif sequences except CgSTP8 (**Figure 2A**). This is similar to the characterization in *Arabidopsis* (Buttner, 2010) and cassava (Liu et al., 2018). The conservation and divergence of motifs in STP proteins may lead to functional similarities or differences among members of different STP families. We also analyzed the gene structure of *CgSTPs* and found that most *CgSTP* genes had three exons and two introns (**Figure 2B**). The variation in the number of exons and introns of the *STP* gene ranged from two to five, consistent with the other plant species, such as *Arabidopsis* (Buttner, 2010), cabbage (Zhang et al., 2019), grapevine (Afoufa-Bastien et al., 2010) and pear (Li et al., 2015a). The diversity of *STP* gene structures may be due to the structural differentiation formed by the insertion or deletion of exons and introns (Xu et al., 2012). Moreover, *cis*-acting element analysis was performed on the promoter sequence of *CgSTPs* (**Figure S2**). The predicted results indicated that *CgSTPs* may be involved in plant hormone (GA, ABA, IAA, MeJA and SA) or abiotic stress responses.

During plant growth and development, sugar accumulation and distribution has proved to be closely linked to the STP members. The most physiological functions also depend on gene expression pattern. In this study, the expression of *CgSTPs* was investigated by qRT-PCR, and it was found that *CgSTPs* had a various expression level in different pummelo tissues (**Figure 3**), which was consistent with the *AtSTPs* in *Arabidopsis* (Buttner, 2010). For example, *AtSTP1* is mainly expressed in the root for the hexose absorption from the extracellular (Otori et al., 2019). *AtSTP4*, *AtSTP6*, *AtSTP8*, *AtSTP9*, *AtSTP10* and *AtSTP11* are highly expressed in pollen and are mainly responsible for pollen tube growth *via* regulating glucose uptake (Rottmann et al., 2018). In here, *CgSTPs* also showed tissue-specific expression, such as *CgSTP4* was specifically expressed in pollen and pollen tubes (**Figure 7**). The further pollen tube growth experiment proofed that CgSTP4 was involved in regulating the pummelo pollen tube length *in vitro* (**Figure 7**). Given the important of sugar content for the fruit flavor quality, the *CgSTP11* and *CgSTP14*, which were highly expressed in juice sac tissue, were selected for sugar accumulation assay. The results shown that the hexose was dramatically increased with the overexpression of these two genes, implying the potential functions in improvement of the citrus fruit sweet quality. Except for these members, there are some other *CgSTP* genes that had lower expression level in detected tissues, like *CgSTP1*, *CgSTP2*, or *CgSTP8*. The more work was required in future to decipher the speculation if they were induced by special development stage or environmental conditions based on *cis*-element assay in their promoter sequence. In summary, the identification, expression pattern analysis, biological and physiological function assays of *CgSTPs* explored the functional CgSTP members in sugar accumulation and pollen tube growth, and also paved a way for further elucidating the more functions and regulatory mechanisms of sugar transport proteins in citrus species.

## Data availability statement

The datasets presented in this study can be found in online repositories. The names of the repository/repositories and accession number(s) can be found in the article/**Supplementary Material**.

## Author contributions

CL and J-HL designed the experiments. WX performed the experiments with assistance from ZL, ZZ, SZ and ML. WX, DG, J-HL, and CL analyzed and discussed the results. WX and CL finalized writing and revision of the manuscript. All authors have read and approved the final version of the manuscript. All authors contributed to the article and approved the submitted version.
