# Supplementary material for: The functional analysis of sugar transporter proteins in sugar accumulation and pollen tube growth in pummelo (Citrus grandis)
Source: Front Plant Sci. 2023 Jan 4;13:1106219. doi: 10.3389/fpls.2022.1106219 (PMC9846575; doi:10.3389/fpls.2022.1106219)
Supplement: Supplementary file 1 [file DataSheet_1.pdf]

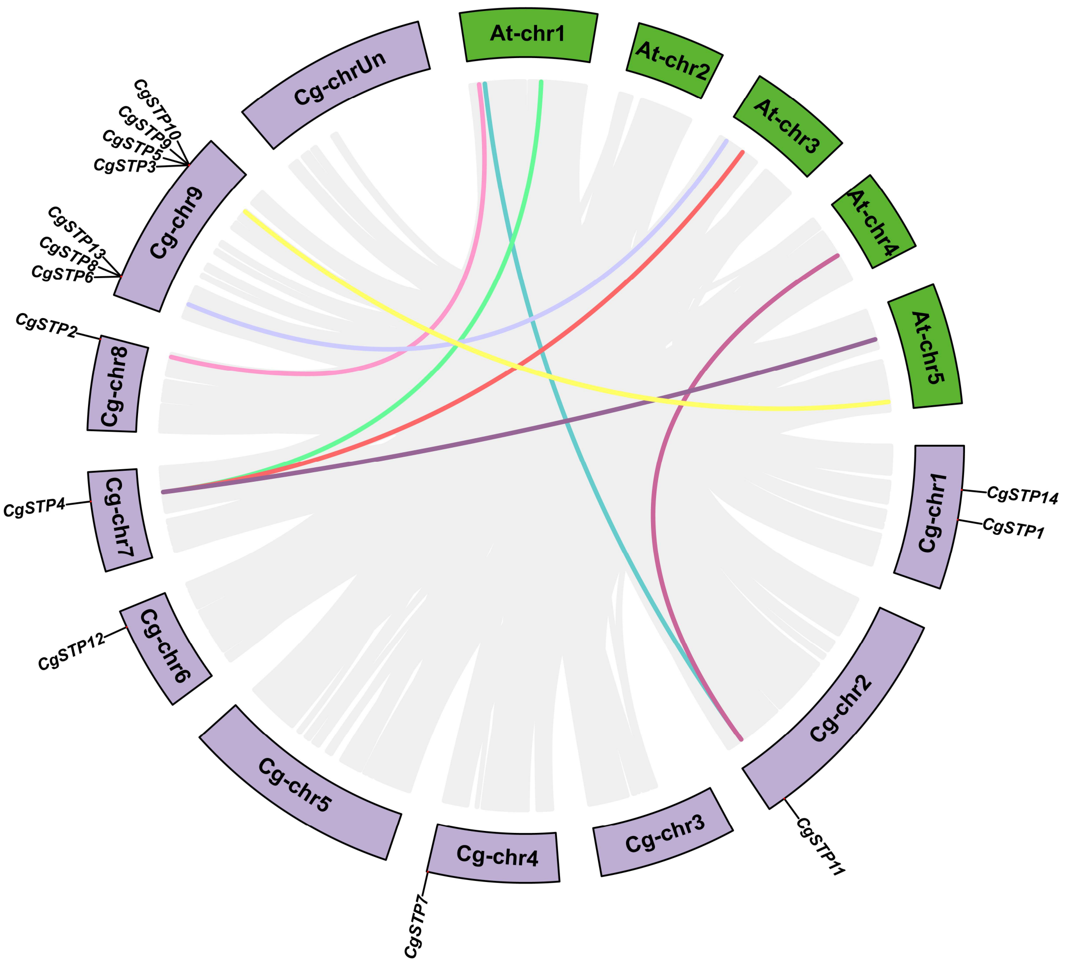

SUPPLEMENTARY FIGURE 1 Analysis of evolutionary relationship and gene location of *STP* family members between *C. grandis* and *A. thaliana*. The chromatic lines were the syntenic gene pairs between *A. thaliana* and *C. grandis* genomes. The chr referred to chromosome. The location of *STP* genes on chromosomes were labeled.

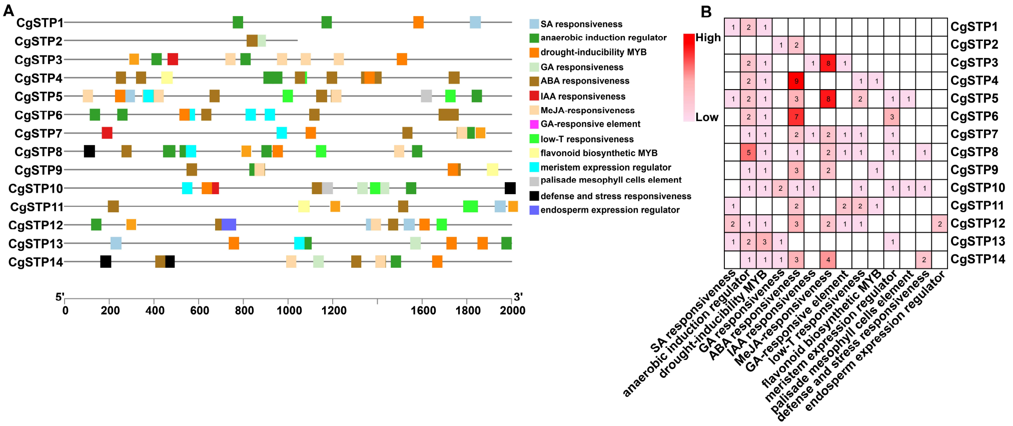

SUPPLEMENTARY FIGURE 2 ***Cis*****-acting element analysis of** ***CgSTP*** **promoters. (A)** The position of *cis*-acting elements in CgSTP promoters. The promoters were uniformly extract 2000 nucleotides from upstream of gene coding sequences. **(B)** A heatmap demonstrated the frequency of *cis*-acting elements on the *CgSTP* genes promoters. The number indicated the frequency of the element. The upstream 2000 nucleotide sequences upstream coding sequence were analyzed via the PlantCARE. SA responsiveness represented the *cis*-acting element involved in salicylic acid responsiveness. Anaerobic induction regulator represented the *cis*-acting regulatory element essential for the anaerobic induction. Drought-inducibility MYB represented the MYB binding site involved in drought-inducibility. GA responsiveness represented the *cis*-acting element involved in gibberellin-responsiveness. ABA responsiveness represented the *cis*-acting element involved in the abscisic acid responsiveness. IAA responsiveness represented the *cis*-acting regulatory element involved in auxin responsiveness. MeJA-responsiveness represented the *cis*-acting regulatory element involved in the MeJA-responsiveness. GA-responsive element represented the gibberellin-responsive element. Low-T responsiveness represented the *cis*-acting element involved in low-temperature responsiveness. Flavonoid biosynthetic MYB represented MYB binding site involved in flavonoid biosynthetic genes regulation. Meristem expression regulator represented the *cis*-acting regulatory element related to meristem expression. Palisade mesophyll cells element represented the element involved in differentiation of the palisade mesophyll cells. Defense and stress responsiveness represented the *cis*-acting element involved in defense and stress responsiveness. Endosperm expression regulator represented the *cis*-regulatory element involved in endosperm expression.

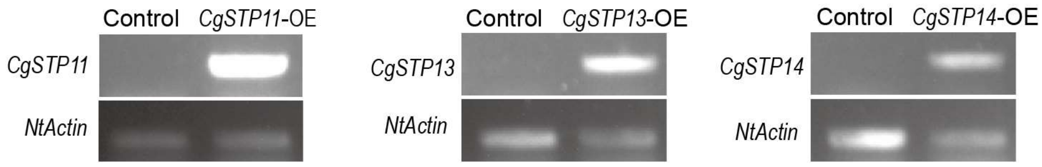

SUPPLEMENTARY FIGURE 3 Expression levels of ***CgSTP11*****,** ***CgSTP13*** **and** ***CgSTP14*** **in tobacco.** The expression of *CgSTP11*, *CgSTP13* and *CgSTP14* was detected by RT-PCR in injected tobacco leaves. *NtActin* was defined as a reference.

SUPPLEMENTARY TABLE 1 List of primers and oligodeoxynucleotide sequences

Name	Sequence (5'-3')
**qRT-PCR Primers**	
CgActin-F	CCGACCGTATGAGCAAGGAAA
CgActin-R	TTCCTGTGGACAATGGATGGA
CgSTP1-qPCR-F	TTCTTGCCTGAGACGAGTGG
CgSTP1-qPCR-R	CTCCGACATAACGGGACCAG
CgSTP2-qPCR-F	GGGACCGATATGCTGGTTGA
CgSTP2-qPCR-R	CCATGTTTGTGCTGACTGCG
CgSTP3-qPCR-F	GTGGCATCAGCGAAGGCTAT
CgSTP3-qPCR-R	TCACTCGGAACCAACCATCC
CgSTP4-qPCR-F	GCAGGACAAGCCATTAACGTG
CgSTP4-qPCR-R	TGAAGTGGCAAAGCATCGAC
CgSTP5-qPCR-F	GAGCATTACTGTGGCAGTCG
CgSTP5-qPCR-R	GTCGTCATAAATGCCACCCA
CgSTP6-qPCR-F	CTCATTCCCAGCGAGACGTT
CgSTP6-qPCR-R	CGGCCTTCATATTGCACAGC
CgSTP7-qPCR-F	GCATTACAGTGGCCGTAAACC
CgSTP7-qPCR-R	TGATGGTAACCCATCCGGCA
CgSTP8-qPCR-F	GGCAAGCGATATTGCACGG
CgSTP8-qPCR-R	AAATACTTGCAGCAGGACGC
CgSTP9-qPCR-F	TGGTTTTTCATGGGGGCCATT
CgSTP9-qPCR-R	AGTAATGCTTTGCCCTGCTGA
CgSTP10-qPCR-F	TGGTCATGGGGACCATTAGG
CgSTP10-qPCR-R	AGTAATACTTTGCCCGGCTGA
CgSTP11-qPCR-F	TTGCTGGATTTGCCTGGTCC
CgSTP11-qPCR-R	GCCGCTGATCGTATTTCGAG
CgSTP12-qPCR-F	CACTCGAAATCCGGTCAGCA
CgSTP12-qPCR-R	AATGGTCATCAGCACGACGA
CgSTP13-qPCR-F	CTTGGTCTTGGGGACCTCTC
CgSTP13-qPCR-R	AACACTCTGTCCAGCTGATCG
CgSTP14-qPCR-F	TGTTGGCTTACGGAAGGTCT
CgSTP14-qPCR-R	AGCTGACCTCATTTCCAAGGG
**CgSTPs YFP Primers**	
CgSTP4-YFP-EcoR1-F	atgggatctactagtgaattcATGGCTGGCGGAGGAGTT
CgSTP4-YFP-BamH1-R	gggggtaccgtcgacggatccAGCAGTCTTGTTAGGTTGGATTTCA
CgSTP7-YFP-EcoR1-F	atgggatctactagtgaattcATGGCAGGAGGGTCTCTTGG
CgSTP7-YFP-BamH1-R	gggggtaccgtcgacggatccTGTGCTAATGCTCTGCTGATTATTC
CgSTP9-YFP-EcoR1-F	atgggatctactagtgaattcATGGCAGCAGGCTTAGCAAT
CgSTP9-YFP-BamH1-R	gggggtaccgtcgacggatccTACCGCTCCTTGTATCTTACTTTCTT
CgSTP11-YFP-EcoR1-F	atgggatctactagtgaattcATGCCTGCTGTTGGAGGATTT
CgSTP11-YFP-BamH1-R	gggggtaccgtcgacggatccCACATTCTTGGTAGCATTGCTTCC

CgSTP13-YFP-EcoR1-F	atgggatctactagtgaattcATGCCAGGTGGTGGATTCTCG
CgSTP13-YFP-BamH1-R	gggggtaccgtcgacggatccCAACTGGGAAACGGGGTCA
CgSTP14-YFP-EcoR1-F	atgggatctactagtgaattcATGGCTGGAGGAGGATTTACAG
CgSTP14-YFP-BamH1-R	gggggtaccgtcgacggatccCTTAACTGGTTCAACAAACTTGCC
**pDR196 Primers**	
AtSTP13-pDR196-F	tcccccgggctgcaggaattcGGACTAGTATGACCGGAGGAGGATTTGCG
AtSTP13-pDR196-R	gggccccccctcgaggtcgacAACTGCAGTTAAAGCCGTGTTGAAGGATC
CgSTP4-pDR196EcoR1-F	tcccccgggctgcaggaattcATGGCTGGCGGAGGAGTT
CgSTP4-pDR196Sal1-R	gggccccccctcgaggtcgacTCAAGCAGTCTTGTTAGGTTGGAT
CgSTP7-pDR196EcoR1-F	tcccccgggctgcaggaattcATGGCAGGAGGGTCTCTTGG
CgSTP7-pDR196Sal1-R	gggccccccctcgaggtcgacCTATGTGCTAATGCTCTGCTGATTATT
CgSTP9-pDR196EcoR1-F	tcccccgggctgcaggaattcATGGCAGCAGGCTTAGCAAT
CgSTP9-pDR196Sal1-R	gggccccccctcgaggtcgacTCATACCGCTCCTTGTATCTTACTTT
CgSTP11-pDR196EcoR1-F	tcccccgggctgcaggaattcATGCCTGCTGTTGGAGGATTT
CgSTP11-pDR196Sal1-R	gggccccccctcgaggtcgacTCACACATTCTTGGTAGCATTGC
CgSTP13-pDR196EcoR1-F	tcccccgggctgcaggaattcATGCCAGGTGGTGGATTCTCG
CgSTP13-pDR196Sal1-R	gggccccccctcgaggtcgacTTACAACTGGGAAACGGGGTC
CgSTP14-pDR196EcoR1-F	tcccccgggctgcaggaattcATGGCTGGAGGAGGATTTACAG
CgSTP14-pDR196Sal1-R	gggccccccctcgaggtcgacTCACTTAACTGGTTCAACAAACTTGC
**RT-qPCR Primers**	
NtActin-F	ATGCCTATGTGGGTGACGAAG
NtActin-R	TCTGTTGGCCTTAGGGTTGAG
CgSTP11-qPCR-F	GTACAGGAAGCAACAGGCGA
CgSTP11-qPCR-R	CAGAGAGGTAGAGTGGCACG
CgSTP13-qPCR-F	CAGGCTTTGCCGTCCTAGTG
CgSTP13-qPCR-R	GCACATTCTTTGTCTCGGGGA
CgSTP14-qPCR-F	GGGCAGCTCTGTACTCGTC
CgSTP14-qPCR-R	TTTCCAAGGGGAAGAGCTCAC
**Oligodeoxynucleotide sequence**	
CgSTP4-s-ODN	G*T*A*C*GGCCGTAAAATGTC*C*A*T*G
CgSTP4-as-ODN	C*A*T*G*GACATTTTACGGCC*G*T*A*C

SUPPLEMENTARY TABLE 2 Twelve different motifs commonly observed in CgSTP proteins

Motif	Protein Sequences	Length	Number of genes
1	WSWGPLGWLVPSEIFPLEIRSAGQSITVSVNMLFTFLVAQAFLAMLCHFK	50	13
2	SCIVAAMGGLIFGYDIGISGGVTSMDPFLKKFFPEVYRKKK	41	14
3	NQSVPLYLSEMAPPKYRGALNIGFQLSITIGILIANLLNYGTAKIKG	47	14
4	NYCKYDSQLLTLFTSSLYLAALIASFVASSVTRKFGRKASI	41	14
5	RGTADVDAEFDDLVRASEAAKQVKHPFKNJJKRKYRPQLVM	41	14
6	ILIPFFQQFTGINVIMFYAPVLFQTIGFGSDASL	34	14
7	WGWRLSLGLAAVPALILTVGSLFLPETPNSJIERGNDEEAR	41	14
8	FGIFFFFAGWVVVMTIFVYFFLPETKGVPIEEM	33	13
9	MSAVITGLVNVVSTLVSIYLVDKLGRRVLFLEGGIQMFISQ	41	14
10	FLAGAAJNGAAQNIYMLIVGRJLLGVGIG	29	14
11	DRVWKEHWFWKRYVGE	16	13
12	VLGBHGELPKGYAILVVILICVYVAGFA	28	13
