# Supplementary material for: The functional analysis of sugar transporter proteins in sugar accumulation and pollen tube growth in pummelo (Citrus grandis)
Source: Front Plant Sci. 2023 Jan 4;13:1106219. doi: 10.3389/fpls.2022.1106219 (PMC9846575; doi:10.3389/fpls.2022.1106219)
Supplement: Supplementary file 2 [file Table_1.docx]

SUPPLEMENTARY FILE 1 The protein sequence of CgSTPs.

>Cg8g023770.1

MPAIALAETGNGKDFPAKLTGQVLVCSIIAAFGGLMFGYDIGISAGVTTMDDFLIKFFPL

VYEKKHRAKEDNYCKYDNQYLQLFTSSLYLAAIVACFFASIVCRKFGRKPTIQAASVFFL

IGAILNCLAQNLGMLIAGRLSLGIGVGFGNQAVPLFISEIAPPKYRGGLNICFQLLITVG

ILAANLINYGTSRIHPYGWRISLGGAAVPALFLLLGSCIIVETPASLIERGKQEQGLYTL

RKIRGVKDVEKEYEEICRATEISNLIKHPYRSLMKKSSRPQLICGTFIHMLQQLTGINVV

MFYAPVLFQTMGYGSNASLLSAVISGTINVASTLVAIVLVDKAGRKILLVQAAIQMIICQ

CAIGVILKMFLLTTNTMPTVPAKVVVILVCVFVAGFAWSWGPICWLISSEIYPLETRNAG

YFFAVSTNMVFTFVIAQAFLSMLCKMRWGIFFFFTGWLLISLIFSATMLPETKGIPIDEM

IDRAWKKHWYWKSYFKNDNHDGSKRTEVAAEIEEKPAA*

>Cg9g005210.1

MVESEDFKSKITVYVVVCWILAAFGGLMFGYDIGISGGVTAMDDFLIKFFPEVYKRKLHA

REDNYCKYDNQILQLFTSSLYLAALFASFVASKVCTKFGRKPTILVASSFFLAGAGISSG

ALNIWMLIIGRILLGIGVGFGNEAVPLFLSEIAPVQHRGAVNILFQLFVTIGIFFANLVN

YGTAKLHPHGWRVSLALAGVPAIFLFIGSIVITETPTSLIERGNEVAGHKALKKIRGVED

VNAEYEQIKLASDIARQVKHPFKELMKRSSMPPLIIGVLLQVFQQFTGINAIMFYAPVLF

QTVGFKNDASLLSSVITGTVNVLSTLVSIYAVDKVGRRKLLLQACVQMFISQV*

>Cg9g005200.1

MAPAIAVGGGDMPVFEGRITVYVVACVIIAAFGGLMFGYDIGISGGVTAMDDFLERFFPR

VYEKKKHAHEDNYCKYDNQFLQLFTSCLYLAALIASFVASRVCSKQGRRPTMQIASFFFL

VGVILTSAAFHISMLILGRLALGIGVGFANQAVPLFLSELAPAKIRGALNISFQLFITIG

IFIANMVNYAMSNVHPYGWRLSLAIAGVPALFLCVGSMSICETPTSLIERGRLEEGRVVL

QRIRGVANVDNEFDSIVHACEMANQVTKPFSKLMKRSSRPPLVIAILLQVFQQFTGINAI

MFYAPVLFQTVGFGSEASLLSAVITGLVNVFSTLVSVYAVDKAGRRALLLEAVVQMFITQ

SIIGIILAIWLKPTGSLNKVEAIIVVILVCVFVMGFAWSWGPLGWLIPSETFPLETRTAG

FAFAVSTNMLFTFLVAQAFLSMLCNMKAGIFFFFAAWIVVMGLFAMFLLPETKGVPVDAV

TERVWKQHWFWKRFMDEEDVKPAAKAPSGIHPHLT*

>Cg9g005230.1

MPGGGFSASVPPAGVEFEAKITPIVIVSCIMAATGGLMFGYDVGVSGGVTSMPHFLKKFF

PVVYRRTLREDDSNYCKYDNQGLQLFTSSLYLAGLTATFFASYTTRRLGRRLTMLIAGIF

FIAGVVFNVAAQNLAMLIVGRILLGCGVGFANQAVPLFLSEIAPTRIRGGLNILFQLNVT

IGILFANLVNYGTSHIKSQWGWRISLGLAGIPAALLTVGSLLVTDTPNSLIERGRFEEGK

AVLRKIRGTDKIEPEFLELVEASRIAKEVKHPFRNLLKRRNRPQLVIAVALQIFQQCTGI

NAIMFYAPVLFKTLGFGGSASLYSTVITGAVNVLSTLVSIYSVDKVGRRMLLLEAGIQMF

FSQTVIAIILGIKVKDHSEDLHTGFAVLVVIMICTFISAFAWSWGPLGWLIPSETFPLET

RSAGQSVTVCVNLLFTFVIAQAFLSMLCHFKFGIFLFFSGWVLIMSCFVFFLLPETKNVP

IEEMTERVWKQHWLWKNFMADDGFDDDEPKKNGHSNGFDPVSQL*

>Cg6g019080.1

MPAVSGIDKGNGRVYPGYLTPFVAITCIVAAMGGLIFGYDIGISGGVTSMPSFLKSFFPS

VYEKQQANSSANQYCQYNSESLTLFTSSLYLAALIASLVASSVTRKFGRKWSMLFGGVLF

LAGALINGLAQGVWMLIVGRLLLGFGIGFANQSVPLYLSEMAPSKYRGALNIGFQLSITV

GILVANVLNFFFAKIKGGWGWRLSLGGAMVPALIITVGSVFLPDTPNSMIERGQHDEARK

QLRKVRGVNDVDGEFNDLVAASEASKQVEHPWRNLLRRKYRPHLTMAVLIPFFQQFTGIN

VIMFYAPVLFDTIGFGNDASLMSAVITGLVNVSATFVSICSVDKWGRRRLFLEGGAQMLI

CQAVIAACIGYKFGVDGNPGELPKWYAFVVVIFICIYVAAFAWSWGPLGWLVPSEIFPLE

IRSAAQSVNVSVNMAFTFLVAQVFLNMLCHLKFGLFIFFAFFVVLMTIFIHFFLPETKGI

PIEEMGQVWKNHWFWSRYVGQDDFLANGGLEMHKEETAVNNV*

>Cg9g023340.1

MAAGFAITSEGGQYYNGKMTAFVVLSCIVAATGGLIFGYDIGISGGVTSMEPFLKKFFPE

VYRKMKEDTNISNYCKFDSQLLTTFTSSLYIAGLIASLFASSVTRAFGRKASILVGGTAF

LAGSALGGAAFNIYMLIFGRVLLGVGIGFANQSVPLYLSEMAPPKNRGAFNIGFQVCVAI

GVLSANLLNYGTQKIKGGWGWRISLAMAAAPASILTIGALFLPETPNSIIQRSNDHQKAE

RMLQRVRGTADVQAELDDLIRAGSISKTINHPFKKIIERKDRPQLVMAILIPFFQQVTGI

NVISFYAPVLFRTIKLSESTSLLMSAIVTGGVGTISTILSMILADRLGRKVLFLVGGIQM

LVSQVMIGSIMAAQLGDHGGFSSGYAYLILVLVCVYTTGFAYSWGPLGWLVPSEIFPLEI

RSAGQSITVAVGLLFTFLVAQTFLAMLCHFKAGIFFFFGGWVAFMTTFVHFFLPETKNLP

IEQMDKLWVEHWFWRRIVGEGVEDSKIQEAL*

>Cg9g023370.1

MAAGMAIASEGGDNNIYNGKITAFVILSCMMAGMGGVIFGYDIGISGGVTSMEPFLEKFF

PEVHRKMKEDTNISNYCKFDSQLLTSFTSSLYVAGLVASFVASSVTRAFGRKPSMLMGGA

AFLAGSALGGAAVNVYMLIFGRLLLGVGVGFANQSVPLYLSEMAPARYRGAINNGFQFSI

GIGALAANFINYGTEQIKGGWGWRVSLALAAVPASILTLGALFLPETPNSLIQRKSDHQK

AKLMLQRVRGTNDVRAEFNDLLKASSTAKTINHPFKKIIQRKYRPQLVMAIAIPFFQQVT

GINVIAFYAPLLFRTIGLGVSASLLSSVFSGIVGAGSTLISMFIVDRLGRKKLFLIGGIQ

MFVSQVIIGGVMAVQLGDQGTVSKGYSILVLILICVYVAGFGWSWGPLGWLVPSEIFQLE

IRSAGQSITVAVSFVFTFIVAQTFLAMLCHFKAGIFFFFGGWVVVMTAFMQLLLPETKSV

PIEQMDRVWREHWFWKKYVGEVDEQGKMEEA*

>Cg9g023330.1

MAVGLAFTSEGGGQYYNGKMTPFVVLSCIVAATGGLTFGYDLGISGGVTSMEPFLKKFFP

EVYKNMREDTNVSNYCKFNSQLLTTFTSSPFIAGLIASLFASKVTRALGRKASILVGGVA

FLAGSALGGAAFNVYMLILGRVLLGVGIGFSNQSVPLYLSEMAPPKHRGAFNIGFQVCTA

IGVLGANLLNYGTQKIKGGWGWRISLSMAAAPASILTLGALILPDTPNSIIQRSNGHEKA

KKMLQRVRGTADVQAELDDLIRASIVSRTVKHPFKTIIQRKYRPQLVMAILIPFFQQVTG

INVISFYAPVLFRTIKLSESTSLLMSAVVTGGVSTIATITSMILTDKLGRKVLFLVGGIL

MFVSQVMIGSIMAAELGDHGGISEGYAYLVLALVCVYIAGFASSWGPLGWLVPSEIFPLE

IRSAGQSITVAVGFLFIFLVAQSFLAMLCHLKAGIFFFFGGWEVVTTVFVHFFLPETKNV

PIERMDKVWREHWFWKRIVGDVAEDIKIEQAL*

>Cg9g023350.2

MAAGLAITSEGGRYYNGKMTVFVVLSCIVAATGGLIFGYDIGISGGVTSMEPFLKKFFPE

VYRKMKEDTKISNYCKFDSQLLAAFTSSLYISGLIASLFASTVTRAFGRKASILVGGTAF

LAGSAIGGAALNIYMLIFGRVLLGVGIGFTNQSVPLYLSEMAPPKHRGAFTIGFQVCVAI

GVLSANLLNYGTQKIKGGWGWRISLAMAAAPASILTIGGLFLPETPNSIIQRTNDYQKAE

KMLQRVRGTADVQAELDDLIRAGSVSKNINHPFKKIIQRKYRPQLVMAILIPFFQQVTGV

NIISFYAPVLFRTIKLSENTSLLMSALVTGGIGTVSAILPMILADKLGRKVLFLLGGIQM

LVSQVMIGSIMAAQLGDHGGFSIGYAYLILVLICVYKAGFGFSWGPLGWLVPSEIFPLEI

RSAGQSITVAVGLLFTSLVAQTFLAMLCHFKAGVFFFFGGWLTVMTTFAHFFLPETKNVP

IELMDKVWREHWFWRKFFDDVGEESKIQGAV*

>Cg1g012330.1

MAGGGFTDAGDLKRAHLYEYRITSYFLIACMVAAMGGSLFGYDLGVSGGVTSMDDFLKEF

FPKVYRRKQAHLTETDYCKYDNQVLTLFTSSLYFAGLVSTFGASYVTRSRGRRASIMVGS

VSFFIGAILNACAVHISMLLLGRIFLGMGIGFGNQAVPLYLSEMAPAKIRGAVNQLFQLT

TCLGILVANLINYGTEKIHPWGWRLSLGLATVPATFMFVGGLFLPETPNSLVEQGKLDEA

RKVLEKVRGTANVDAEFSDLIDASNAARAIKNPFRNLFKKKNRPQLVIGALGIPAFQQLT

GMNSILFYAPVIFQSLGFGSGAALYSSVITGIALCIAALISMAFVDKFGRRAFFLEAGTE

MIIYMVIVAITLALEFGEGKPLPKGIGIFLVIVICLFVLAYGRSWGPLGWLVPSELFPLE

MRSAGQSVVVCNNLLFTALIAQAFLAALCHLKFGIFLVFGGLVVIMSAFIYFFLPETKQV

PIEEIYLLFENHWFWKRIVKEDNGKFVEPVK*

>Cg1g019290.1

MPAAGGFDKGNGKGYPGKLTPFVTVTCIVAAMGGLIFGYDIGISGGVTSMPSFLKRFFPS

VYRKQQANSSTNQYCQYNSETLTLFTSSLYLAALLSSLVASSVTRKFGRKKSMLFGGVLF

FAGALINGFAQGVWMLIVGRLLLGFGIGFANQSVPLYLSEMAPYKYRGALNIGFQLSITI

GILIANVLNYFFAKIKGGWGWRLSLGGAMVPALIITIGSLVLPDTPNSMIERGQRDEARE

KLRKIRGGNDVDEEFNDLVAASEASKQVEHPWGNLLKRKYRPHLTMAILIPFFQQLTGIN

VIMFYAPVLFNTIGFGNDASLMSAVITGLVNACATLVSIYGVDKWGRRSLFLEGGTQMLI

CQAIVAACIGAKFGVDGNPGELPKWYAVVVVLFICLYVAAFAWSWGPLGWLVPSEIFPLE

IRSAAQSVNVSVNMAFTFVVAQIFLNMLCHLKFGLFIFFAFFVLVMSIFIFFFLPETSGI

PIEEMGQVWKNHWFWSRYVGEDDFVSNGGVELQKGSNAINNV*

>Cg7g013990.1

MAGGGVVVQGGAKNYEGGVTSFVLVTCLVAAMGGLLFGYDLGISGGVTSMDQFLKDFFPK

VYRKQLNKGHESAYCKFDSQLLTLFTSSLYLAALVASFFASVVTRMYGRKMSMTVGGISF

LIGAIINGAAVNIAMLIIGRLMLGVGVGFANQVVPVYLSEMAPAKVRGALNIGFQMAITI

GILIANLVNYGTAKISGGWGWRVSLALAAVPAILMTVGSFFLPDTPNSILERGHVDEAKK

MLQKIRGTPNVDEEFQDLYDASEAAKQVHHPWTNILRGRYRPQLTMCTLIPFFQQFTGIN

VIMFYAPVLFKTIGFGAEASLMSAVITGVVNVVATLVSVFSVDKFGRRILFLEGGVQMFI

CQCLVGIMLALKFGLRGEGTLTKFEADFVLFLICAYVAAFAWSWGPLGWLVPSEVCALEI

RSAGQAINVSTNMIFTFVVGQVFLSMLCHFKFGLFFFFAGFVAVMTVFVFYMVPETRNVP

IEEMNRMWKAHWFWGKYIPDEAVIGSSNEIQPNKTA*

>Cg4g024730.2

MAGGSLGPAGVAKERAEQYQGKVTPSVIVACLVAAIGGSIFGYDIGISGGVTSMDAFLKK

FFHDVYLKKKHAHENNYCKYDNQGLAAFTSSLYLAGLVASFVASPVTRDYGRRASIICGG

ISFLLGAALNAAAANLAMLLTGRILLGVGIGFGNQAVPLYLSEMAPTHLRGGLNMMFQLA

TTLGIFTANMINYGTQKLETWGWRLSLGLAAAPALMMTVGGILLPETPNSLIERGKKVEG

RRVLEKIRGTKEVNAEYQDMVDASELANSIKHPFRNILERRNRPQLVMAIFMPMFQILTG

INSILFYAPVLFQSMGFKGDASLYSSAMTGAVLASSTLISIATVDKLGRRALLISGGIQM

ITCQVIVSIILGLKFGPNQELSKSFSILVVVVICLFVLAFGWSWGPLGWTVPSEIFPLET

RSAGQSITVAVNLFFTFVIAQIFLTLLCSFKFGIFLFFAGWVTIMTIFVYFFLPETKGVP

IEEMILLWRKHWFWKRIMPVVEETNNQQSIST*

>Cg2g041230.1

MPAVGGFDKGNGKEYPGNLTPYVLVTCIVAAMGGLIFGYDIGISGGVTSMPSFLKKFFPS

VYRKQQANESTNLYCKYDSETLTMFTSSLYLAALLSSLVASRVTRQFGRKLSMFFGGILF

LAGALLNGFAQAIWMLIVGRLLLGFGIGFANQSVPLYLSEMAPYRFRGALNIGFQLSITV

GILIANVLNYFFNKIHGGWGWRLSLGGAMVPALIITVGSMILPDTPNSIIERGRHEEARE

ELRKVRGVNDVEEEFNDLVAASEASRQVEHPWKNLLQKKYRPHLTMAVLIPFFQQFTGIN

VIMFYAPVLFNTIGFGSDASLMSAVITGIVNVVATMVSIYGVDKWGRRFLFLEGGVQMLI

CQAVVAACIGAKFGIDGNPGELPKWYAIVVVLFICIYVAGFAWSWGPLGWLVPSEIFPLE

IRSAAQSVNVSVNMLFTFLVAQVFLNMLCHLKFGLFLFFAFFVLVMSFFVYFFLPETKGI

PIEEMGRVWKTHWFWSRYVGEDDFVPGGNVEMHKGSNATKNV*
