# Supplementary material for: The functional analysis of sugar transporter proteins in sugar accumulation and pollen tube growth in pummelo (Citrus grandis)
Source: Front Plant Sci. 2023 Jan 4;13:1106219. doi: 10.3389/fpls.2022.1106219 (PMC9846575; doi:10.3389/fpls.2022.1106219)
Supplement: Supplementary file 3 [file Table_2.docx]

SUPPLEMENTARY FILE 2 The promoter sequence of CgSTPs.

>Cg1g012330.1 - Up_Stream_Len 2000

ATTGAACATACCGAGGGATATACACATACATATAAATACACATAATAGATTTTCATACATGAATCTTTAACTATATTAAAGTAAGGAATAAAACAACTATACTAAAGTTAGGAATAAACTAAAAAAATAAAAAAAATACAAATTTTAATACACTGCAGAACAAAATACATGCATTGGAGTTTATATTTTTTTATCTTTTACTTTATCCGTGATTTTATATAGTTAGGGATGATTGGAACACACTCATATATATAATTTCCAATCAGGGACTACCGACTATATTAAAGTCAAAGACAAGTTAAAAGACAAAAAAAAAATAATTTCAATATATATACTTTAATAAAATGTGAGCTCACTCAATATATATATATACTCTCATACTAATGTCCTCATTTTTTTTTATGTAGATATGTCCTTTTATATGTATTTAGTTTACACACGACTGTGAATGCGTGTTATTTCAGAGATTCAAACCTCACTCCCAGTAAGTAAAGGGTTGTTGTTGCCAGTGGACTAGAGGGAGGCTTAGTGGCGTCTCTTAATGAAATTTGACAAAAAGATTATTTCAAATGAAAGCCAAAGCAATGGTCGATCAAGAATGATGAAAAAAAGTCTCAACTGAATCGCTTTCACTTACAGCAAGGGGGTCCATATGGTCCCATTCTAATATAATCTTTCTATGGAAATTTGGGGCCTAAAATATTTGATAATTTCTAATTTATTGACCAATGTAACATACATCAAGACATTAAATCCAATACAAAATAAAGAGCTTTTGTCTTCAAAGGGAGCTGTGGGCCATAAACTGTACACTAATGGCCAAAAAATAAACCGGCGAAAAAAAAAAAATTCTAAACGATCCATTTCATGCTTGACTTGTCAAACTCTCTCTCTCTCTCTCTCTCTATTTGTAATATACAATGGAGATATTTGCAGAATATTAATTCAAAAATCTATTTCCATAAATTTTTCTTATAATTCATTAGTTTTAATATAGAGAATCAAATAAGTCATGTGAGGCTCGAATTAATCCCTCAAAATCATTCTGAGTTATGGTTGATTTTGTGAGTGTAATTCTTCAAAATCATAAAGTTTATACCAATTAATACACGTTTAATGATTTATTTCTTAAATAACTAAATTCAATGTACAACAATTATTGGACACAATTTGTTTTTAACTAAACTTTTATCCAAATTAAATATACATGACAACTCATAGAGTCAAAGTCAATGAGGCATTTTAATCTAAAATATGGGGAACACAACTTGCCATTTATGATATGAAAGTAAATATCCCTACCGAGATTATTGGGATAATTATATTTTATAACAACTAGCATAAAAATAGTTGGCTAAAAACAGAGAACCGAAAAGGAAAAAAAAGACCGAGTCAAAAAAGACTAATTATCCCAATAGTTAAAGGCTGAATCATTCATCTCTATCCAGACGTTAATGTTGTTAATTGATATTAATTTTAGAGGATTTGGATTAAGTTTAGCTACTAATAAAATTATGAACCATAAAAGTTGGTTTTGGATTAATATTACCCGAGAATGCAGCTATGGATCAGGATAATTAAAACAGCTTATATAATTATGGCTGGGGCATTGTAAATTTTAGCTGTACAGAGATTATTAACAAACTGGGTATGTTTATAAAAGCCAATATTGAGTATTAATCCAAATACAAGTCTCTGTCACAGTCACTCCCTTCTCATTTGAGTTCCAGGTAAAAGAAAAAAAAAATATTGATTTTTTTATGATTAATATTATTGATTAGTTAATTATTTCTTTATATGAAAGGTGACCTCATCTGGGTTTGACTCTTTTTGGCTATTTATACATTCCTTGTACTTAACAGTTCGATCACTCATTGTTGTGTTGCTTCGTTTTGTTTCATTTCTTTTTTTTCAGAATATATATGTAGCACAAAAGAGAAGGGAACAAAAAAGAGAAAAAAAGTTATTTTTGAAAGAAAACAAAACGCAGAGATTAAGAG

>Cg1g019290.1 + Up_Stream_Len 2000

AATAAAATATTAGTAATAAAAATTATAAAATTTCTACTTATTAAAAGCTTATTGTATATATGTTGCATTGAAATAAGTTAGGTTTAGGAATTAGAAACTTTAACTTAAATATATTTATGATTAATTTTTTTAATGTAATTTTTTTGATTAAAATAAAAATATATAAATAATTAAAATAAAGAATGCGGGTTTGTGGACACTTGCGCTGGTACCCAGCGGATTTTTTCTCCAAATCCACTCGCAACCTACAGTAGATTTAAGTTTGAAATATCAAACCCACTTGCAACCCACAAAATTACTCGTAATGGGCGGGTTTTTATGGGTGAGTTTGAGCGGGTTTACTGGTTGGCAGGTAATTTTGCCATCTCTATTGTGTATGGAATGAAATATACTCCTTGAACTCAAATTGATGCTTAAAAGAAAAGTAGAAGATGTTTTAGGCTTTTCATTTGAAAGGGTATTTTTGGCATTAAATAAAGTATGATTGAGGGGTTAAAAAGAAGCGGGGGATGTTTAGTTCAATTTTGGAGGGATGCCAACAGTGCAACTCCCTTTTAAAACTCTCAAGAAACCCTATTTCATATTTACGTTTATAGCTCTTCTGTTATGGAATTAGAAAAAGGACATAAAATTAAATACAATAGACTTTTAACTAAACCTTACTATCATTTTGAATAAATTAATTAAATTGTAAATATATTTTTCATGATAATAATATCATTGTTAAGTAATTAATTTATTTATTATTCCAAATAATTATATTTTATCAACAAAATTAATTACTAAAGGGGTGCGAAAAGCATTACTCTAGAAAAATTGTAGCCCCAACATTTTAGTAGAAAATAAACAAGGGAAATTTTTTTTGAAAGAATGTGGTTATCAGATTAATAAACAATTTTTTTTAATTATTGATGGAGATGCCAAAAGAATTTTATACTGTTTTTAATATTTTAATATGATAATTAAAAAAAAATTAAAGTGTTCATCCCAATCCAGTTCTAAACTTATCGTTATCTATTGTAGAGCTAATGAATTAGCAGTGACTAATCTTTACGGTTATAAACGAAAAAAAAATATGTTTGAATATATCATGCAATCTATTTTGGTCCAAAACGACATTGATTATCTCAGTAAAACAAAAAGTTATCCAATGAGAGATTGTGACTAAGTCAATGTCTAACAGCATCTCCAAAAGGGTCTTTAAATTTTACTCTTTAAATTTCAATTTGCTTACTGATGTGGCAAATAAGTGTATAAAAAAATATAATTCCCTCCAAAAGACTCACCAAATAAGAATAAATTAATATTATTTTAATCAAATCTTTCCCACTATCAAATTGAATTGTTATTATATTTTTTAAAAGAAACATAAATAATAATTATTACAGTCTTATCTTTTCAATAAATAATAATAAAATATTAATAGAAAATGGAGAAACTCACTCGGCAATATTAAAGAGTGAGAAACAAATCTTATTTGAAGAATCTAAATTAACGTGTCTTTTAGAGTGTTTAATATTGATATGACTCTTCAAATAAGAAGTAGAATCTTAGTGGGAAAGATTTGATTAAAATAATATTAATTTATTCTTATTTGAAAAGTCTTTTACAGATGCTTTAAGCATCAGCTTTCCTCAGCTAACGAGAGATCGAAATTGAAAATCTAACTCAAATAATGTAGATAACTAAGAATCCAAATCATAACTAAATAAGAAATCCGAATTAAACAAAATAATCTTCTGTTCAGTGCGTACTAGATATTAACGGAGTTGTTGTTTAATAAAATTTCGGATAAGAAAAGCAATCCGAAAACAATTAAAATTTTATCTATATAAAAAGCAACGTAACTTCTTCTTATGCTAAGCCTACGAGCTCTTTAATTTAGTTCAATTTTTTTCTGCTTTCTTTGTTCTCCGTATTCATCTTCCATCTTTCTGTTTTGTTCTTAGAATTCTTCTTAAAATTAAGAAAAAAATAAAATAAAACAAAAGGCTGAAACT

>Cg2g041230.1 - Up_Stream_Len 2000

AATTTTACAAATAGAGCCTCCGGGACCCAGTCAAATAGTTTTTTAAATCATTGTATTTAACTGGTCAATTGAGTTCGAATTATGAAATAGTAGAAAGAAAAAAAAAATGAGTTCGCTTTGCACTGATTAAAAAATAAAATGAAATAAAATACCACAAGTAAGTAAGACAACTCTGAGCCGATAAACAATATGAAAATTAATCCAAATATGAGTTAATGACTTCCCAAATGGACTACATCTTATTCTGCTATAGACAATGCAACTCAAAACCTTACTTAAACTAAAATGACAAATACAACTCAAATATGAAAATCAAATAGATTATCTTTGTTAATTCCTCTACATGACTATATGTTAAGTTGTTAACCATCATGATTACGAATTACTGATCTTTTTTCTCCTTTAATACGTGGGGTTGCTACAAATTATTTTTATGATAAAGTTGATTTTCTAACAGAATTTGTATAAACTAGAATAACTAGCAATGATATAATAAGTAACAACTAAACAAAGTCAAAGTATTCTCTCTGTGTGTGTGTGTGTTTTTTTTTTTAAGATTATCTAATAAACAAAGTATTCCCTTGTTTGATTTTCAAGTTTAAAATAATTTTATTTTAAGAGAAGTAAACAACATGCTTCTCTAGTTATTACGAATCTTCCTATATGGGCGGAAGATATTTTCTGAAAAAAAGAAACAAAATCATGAAGCGTTGTGAGAATCAAAAAGATTCTCGTTTCCATTAGTTAAAAAAAAAAAAAAAAAAAAAACATTCTGACCGATAACCTCTACGGGCAAAACTCAGACCTGTGGTCGACTGGTCGTTGTTTGGCTTAAAAATCGCAAGCATCTACCCAGTCATAAATATAGGGTAAAGACAAATCAGAGGGTAAATTTGTCAGAAAAAGTAAGCCAGTTCAGAATCTCTTTTATGGCAACCTTTACCATATCCCGCAAGTCCAGGAATTGACAGCCAAGCAAAAAGCCTTAGAATACGTCATCTCGATCCTTCCTAATAAAAACCCCTCAATTTCGATAAATATCGGAATTGCCATTTCAACCGCTAAGTTGCTCGTTATACTGAAATTAATCGGGATTTTTTTTAATTAATTAATGTGGGATACTGCGATTCGTAATTCGACCTTAAAACATGCCAATAATTACGTAAGTATTATAAGAAAAGGACTTTAAACTACCGTCTCTCTCTCACTTGGTGTTTTATACCTGGTATCTTTGATATATCACATGGAAGTATGGAACAACTTTGCTGTAAAGAACTTTTAGTTCACGTTTTATCAAAACTTTTAAGACACCTACAAATCCAACGTTATCCGATTTCAGATAAAGCAGAAAATTAAATTAGGTGAACTGGCAAAATTTAGTCGACTCACTCCTGACGTGTAAATTTATTTCTAACTAATCGTTATTTGTCACCGACCCTTTATTTTCTATAACAGTAATGATGGTTTTGTATTGGTAGATCATTATTTCCTTACAAAGAAAAAAATTAATTGATTCCCATTACTTGAATTTTTAATACCAGCGTCCCCACCGTCCGATGTGAAACTTAATTTGTTAAGTCGATTTTGTAGGACCATCCAACGGCGGAAAAGCGAAGGAGACTTGGGGACAAAGAGAATAGTTCGTTCAGTTGTTACTTATTATATCATTACTCGTTTACTCATATTCTGTTAAAAATCATCCTTATCAGAAAAATAATTGGTGACACCCCCCGCATATTAAGAAATTAGAGTTGATTCACAATTAAGATAATAATTATAGTTGACAGCTCAACAGTCGTCCGCCACCTAAGAAAATAGGAAGACATTTCTCTATATATACACTTGCCAAGCTTGTCTAAAAGCTCTGTATCTTCTTTCTTTATTGCCTGCTTACTCTTTAGTCTCCAATTTTCTTGTTTATTTTCTTCTTCTTCTTCTTCTCCGTGTCATCGTTTCTTTATCTTCTTAGAATCAAGAACAAAAAGGAAAAGGCTGAAAAA

>Cg4g024730.2 - Up_Stream_Len 2000

ATATCCATTTATTAATTTGTTTTGAGAAGAAAGAGAAGTTGCATAAGATAAAAAAAAAAAAAATTGCTTACTTTTAAAAGTTACTATTAGAAATGGAGAAACATATGAAATACTTAGTTTTTAAAAAAAATTACCAAACATTGTTGGGCTCTAGTAGATGAAGAAAAGAAGAATCAATTATATACGAGTGATTGATTAGAGAAACAGAAAAAGATGTATTAAAATTTTGAAACCTTCAACTTCATCTGATATTTAACAAACAACAAGGAGTGCATTGTTAATGATCAAATAAACACATCCACCATACCATTGAAGCTTTTGCAGTAAACGTAATGGTGACAGCAAAAAAAGGCAAGATGAAGAATATGGCGCTCCTCATTGAGTAACCTCTAACTATTCTCTTTGAAGTTTTAAGATTGTGTCACTATCAAATCTAGAGAATTGTAGAGGGCAACAAGTAAAGCCCACCCACAACCCTAAAATGGAAAAAAAAAATTGAGTAGAAATTGTGGATCACTACTAGTGATTCAGAAACAAACAACAATGTCATTTGTGAGAATCAGCATGAGACGCACATATGTACATACAACACAAGAACCGCAAAACAAAGTCATTAAAGTATGCACCAACCAACCAAAAACAAGACATGAATATTTGTGAAATCAATATATCAAACTCAATTTTTTTTTTAATACTGATTAATCATCAGATTACTGCATTACACAGATATTTACAACAACTGCTATTGTAGCACAGATATTACACTGATATTTACAATCAGATATATATGATTGAGACTTACATTATTACATTAAATTCTATGAAGTACATGCCCAGACAAATAACCCCTCACTGTAGAGGGATGTCCATACTACAGGAGAGGGATGCCCATACTCCAGGAGAGGGATGTTCATACTCCACTTGCATTTCGCAAGGGAGAATGATTTTTAAATATAAAGAACATTTAGCCCCCACAATGCTTTTGTGGGGGCTAGAACCACTGCATCCCACCACTTCGGCCAAGGCCGAAGTGGCATATCAAACTCAATTGGAAAAACACAAAACCATCCTCAGACCCTCACTATCAAAATATCTCCCAACGGTCCGTCTATATAGACCATATTTGTGCAATCAATCAATATATCAAACTCAATTTTTTCTTCAGATGTACAACTTTCAATAGATTTGAGGAATGTAATCATAACTTATAATATAAAATAAATAAAAAAATTAATAACATGGGTTTACCTACTACTAGTTTAATTAATTAATTAGTCATTCAAATTCCAATCTTAATTATTTATTATTATTATTTTTTCATAACAAGAATTTATAAGAAGGCACACTCTTGAAATGCATCATATTAATAAGGTTTTTTTTCCCCTTATAAAAACACTTTCAAATATTTTTGTGTATCTATTTTTAAGAAAGCACTCTTGGAACACAGTTTCCTATTTAATCAATTTCTTTTTAGTAAGAGATTTGGTGTAAAATGTATTCACTGTCGTTTTTGTCTGTTTCCTAGCAAAACGGTCCCATTTAATTACTTGGGATAATACGTATTTTCATTTTCCTGGTTGATTTCTTCCCTGCATTAATAAGGCTTTCATATTCGCTCACTCAATGGCTGCTTGCTATTGGAAGCCTTATCAAATGACAACAGTTTCTTATTTTGCAAAATTAAAAAAAATAATAATTATTTACCAGAAACAGTTGTCATTTGTAACAACTATGGCAATAATCTAGATAATTAATAAAGGCACTTGCAAAGATAAATATATGCATATGATATGTCAAATGTCATACAATCTCTCTATCTATCTATATATATGGAGCTACGAGACTTGAACAACTGGTCCTGTATAATAGACCCCCAGAAAAAAAAAAACAAGTAATTTTCAATCTCATGCCAGTTTGAGCGAGGAAACAAGCTTTTGTTTTTTAGTGTTTTGTGTTTGGTTTTTGAGAGGACAGAAAGAAGGAAGAAGAGGATCTTGAAA

>Cg6g019080.1 + Up_Stream_Len 2000

ATCGTGCATAATCATCTAGCATGGCTCTCATTGTCCCTCTGCAGCGCCGACTTTTTCCATTATTTTCTGCCCATCTTCACCTTCGTCATGAACCGATCAAGACCGAATGGATCAGCTTCTTCAGCCTCCTTCTCAAACTTAACCGGTCCGTCCCTCGTACCAGACCTCTCAGATGTACCTGCAAATGCCTTGTCGGGTTTAAACCGATCAGTCTTTCTAATCTTCTCCAACTGCTCATCCGCATTTGCTCCATAAATTTCAGCATCAACATCTTTTTTGGGCCTGTAAAGGGTTGAAAGAGTCGGCTGTGCTGTAAACAAGCCTTTGTCATACACATTATACTGATCACCGTCAGTGGCAAAACCAGAGTCCATTCCATTCTTCTGATTAAAAAGCCTTGCGTCATAAGTAACCTCACCGGCTCTGCCTCCTCCACCAATAGAGGCCATCCCAAGGGCAACTTTCTCGCTAACATCCCGATCTCTGTCTCTAACAATCTTACTATTCTTCTTCCCCATAGCAACATCCTTGGCCAGCAAATTTCTCTCCCTCTTCCTCTCGCGACGTCTCTTCTCATGAATTCTCTCCCTCTCCCTTTCCCTCTCCCTCAGCAATCCCTGCTCGCTTTCCTTCAATGTCTCCATTGCCTTACGTGCTAAATCCCGTAGCTGCTGCTCTTTCCTTTCCTTCTCCTTCGTGAGCATCTCCATCTGCACCTTGAATCTTATTTCAATGGCCTCCCTCGCCTTCTGCTCTGCCAAATAAAGAGCCTCCGACAATTTAGCAAATCCCTCATTTACCTGAACCTGCAGAAGCCCTCTTCCTTCGGCAGCGAGCCGCTTATCCAGCGGAATTGTAAACCGCTGTGGGTTCTTCCAATTTGATATACATGGTGGGATTCTCCAATCCAGCTGATCTTTCACAGTCACAGGCCTGGGAGGGGAATGCATGACCGGTACTGGCAGAGATCCCGAAGCTTTCGGCACACGCTTGTGCTTCAACTTTGGTGGCTCAAGTGGGTCCAAAGGCATCGCCACCACTCTTAAAATCCTCCTTTTAGCCCCAGAATAGAAGTCCACCGATTTCTGTGATGGTCTGTACATAAAAAACTTGGACTCTAATGACTGCGTAGGTGCAGCATTCACAACACTGTTAACAATCTTTTCAAGTTCAGCTTTTGTTACAGGAGTAGTCACGTCAATCTCATCTTCTTGATCAGGAAAAGGAAAAGCGCTACCATCATCATCATCGAAATCCTCAAGTTTCCGAGGAAGTATTGCCATGTTACAAAGATTTCCTGCTTGTCAATCAAGCTTGTGATTAGTCCCAAGCAATTTAAGGGTTCCTCAAGGGCTTCTTGTGTTTTTTGTTATCGTACACAACCAGCAAATTTCTCTCCCAGTAGCGGCGCCTCCGTTTTGCGATTTGCTAATGCTATGCTAGCTTGCTAACTTGTAGCGGAGGTAATCTCACTAATGCTAAAACTAGATATCCTGATGTAAAAACTTTAAACACAAAAAAAGTGCTCGTTTTGAAAAATTATTTGAGATTACAACCTTATTTATTAGTTTTAATTATTACTCAAAACAGCTATTACAATAATTGAAATCCTAGTCAAAACTAAACTAATAATAAAACTAATTAATATGGCAACAAATAAGACAAATCCTAAACAAATTAGAAATACCAATTAAAAAGAGGAAAATTTCGGATTAGAACAAAAAGAAAGGGCAATAATCTTTATCCGTATCCGTAGAAAATAAGGAAAATTTTTATGTAGATAATAAAGACTTGCCGACGGTAGTGTCCTAAGAAAGTAAGGAAAACCAATCTATATAAAGAGACTTGCCTGTGCTTGTCGAAAAGCACTATACCTTCTTCTTTTAATTAGCCTGCTAGCTAGCTCCTTAGTAATCAGTCCCAAATTCTCAGCTTTCTTAACTGCTTCTCCGTTACCTTATTCATCATCTTCATTAAAACAAGAAAACAGGAGAAAAAAA

>Cg7g013990.1 + Up_Stream_Len 2000

CCATTTACTGACATTTTTTATTCCCTAGCTTATAAAGTCTGCCCTCACAAATGCCATGAACACCAATAGGAAATACAGTATTAGCAGATTCAATAAATTAAGACTTGATTTCTGTGAGTTAAACCATATGGAAACTTGAAATGAAAATGCAAGTAATGGCAAAAAAACTATCAAAATAGATAGATGCGATTCAAATAGATCGAAAGCAACTTATATATCAAATAATATCTCAACCAAGGCAAAACACTGAATTAAGTTCTGCATGATGATTAATGACAAAAGGAAATGGGTGCTCCGCTCATGAAGCTCTCCACAGTTTGTTTACGCATGACATATATAGATAAGTATTATTGAATGACTCCTCTAAGCTGTATGAATATTTTTGTTAGAAGTAGTTAGATGATTTACAATTTTACTATATTTCTCTGTGATGATCTAGATTAGAATGAGTTAAAGAAAATAAAGTGTTTGGTTGAAAGGAAAATTCTAAAGCTTCAGAGAGAAAATATATCAGACTGAATTCATTTTATTAATTCTTAAACTGAAATGAAGAATTACAAAGCTTTATATACACATGTCTAGAAGATTCTACAGCAACCAACGCATGTGAATAATCTCAATAACTTATAACAAACTATTGGCGGGAATCATTAACAAACTCTAAGAGTTGTTTTGCACGTGTAACTAATTGCTGATGTGGTCAATGACTCAACAACCTTTGCTGACTCAGCTGATGACATGGACATAAGGATTTATTCTTAACAGAATGTTATTGTATTATTGTAATTCAATAGTATTTATCTGGATATTAATTGGTGTGAACTAATGAGCTATAATTGGAGTTACACAATAATAAAGTTATGTTTGTATAAACTCATGAGAATTGATATAGTGATCGTCGACACTGATTGGGAGAGCTATGGTGAGTGATATCAGTAGATCAATTTCCTGTAAACAAATCACTATTGATGTAGGATGAGAAGGGTACCTTTCAAAAGTTATAACTTGAGTTACACAATTTTGCCCTATGTTCAAGTCAGAAATTTCATTTTTTAGTTGTTTTGCAATTTTGAGATGTTTTCCTAATTTCTCTTTTGCATTCATGATTTTTATCTTTTTATTTTTAAATATTAGATTTGGTGCTATTCATTTTGTTTTATTGAAGAATTTTATTCTATTAAAAAAAAGATTAAGAGAATTTTATTATAAAATAAATATTTTATATTTACCTATTTTGATAAATAAGCGATATTTAAGTCTACTATAAAAGAGTCCTAAAACACTCGTTTTGTTCATTCATACTTTTCAATAATATTTTCATTATTCAGAAACTTTATTTCTCTTAGTTATCAATCTTTTTACATTTCTACATCGTCAATTATTCTATTTACGTACAGTGAGTTGTTACAAACTTGTTAATTGGACTAATTTGTAGTTCCAAAAACAGAAACGTGATTTTTAAATAATTATAGCTTATCAATTATAGTTAGAGTGATGCCTAAATCTTATAACGGAATTAAAAAAAATGGAATTATTTAGAGAAAATGGAAAACGGCCGTTGATTTTACATAGCTGAGTTAGATGTGGTACAGTTGAGATTAGTTACCTATTTTAAGAAATTCAATAACATGAAACAAGGATAAGCCAACCGCAAATTCACATGGAACCGAAAAATAGCAGGCATTTGGATAAATTCTTGATTGATATATGGATAAAAAAGCCTGGTGGATTTAGACAAGTCTTATTCATAATTTCCGGACAGATCTGCTGAAATAAGATGTCAAATATAGAGAAACAACCACTTGGAAATCTTATATAAAACGAGTTAAATATCATGTCCATTAATTAACAGGAGTTTCTTTCTCAGGTGCTTATGAAGGATCTATCAAGGCAAATAAATACCTTGATAAATCCCTCTGCATCCGCAGCGAAGAAAAAATAATTAATAAACTCCTGTAACATTTTACCTACAAAATTAAAAGCAAAAATTTGCTGCAAAG

>Cg8g023770.1 - Up_Stream_Len 2000

AACCACTAACCATAAAACAATTTTATTAGTTCTTCTTAGTGAACATCGAACATTATGTCCTCCAGCATTGCTTGTTATTTAAAGAACTGAATTAATGCACGCACCTCATGTGAAATTGAGCAGGAGACCTCAAAATCTCCAGCGCAATCCACCAGAGAAGCCGACCTCACTAGCAGCAAAAACTACTCAGTGGAGAATGAGAAAGAAGATAAGGGGAAGTCTGTCGTAGGCTGTGAATGTGAATCCAACAATGCGGTGGAGAATGAAGCAGCCATTGAACCATCAGTACTAGCAGCAGCTGAAACCGCTGTAGAAAAGGTAAAATAAATTAAGACAGCAGGAGACAGTGAACGTTATTGGATTCAACCAATAAGTTTATTACCATCCCTGTCATAGCATCTTCTTTCCTTCTTGCTTTATAAGTTCAAAGTGTTGTTTCATTTACGATAATTGAGTTTTTTCGTATTTTTCCATCTGAAGGAAAGTTACATTTACATTTTCATTCTCTGTGTCATCGGATGCATAGGAACCATCTGACCCTCTTTCGCAGAAACCTGAAGTAGAAGCTGCTGTTGTGCACAATGTGAATGAAACAGTAGCTGATGATACAAGCAACGAGAAAAGCTTGATTCATCAAGACAACGTTTCTGACCAGTTAAAGACCATCGAAGATTATAAACATGAACCAGATGATACTAAGGTGGTTGTAGAACCATTAACATCAATAAAACCTGAAGTCAAGGAAGCTCCAATGAAACCAACACAGCAGAATGAGGTGAATAAAGTCATTTTCCAGTCTTGAAGCCAGATGTTCAAATGATGGGGGTTTTGTATTCTCGAATCAAGCTAACATATTGAAACTGGCCCATTTTTGAGGACAAGGATGGAGCCTCTATTGCAAATATACTAATTTTTTGCCAGACATACTGAGATAGTTTTTTTTCCCCTTGTTTTTAGTTTCTTCATAGTCATAGAAGCTTTGCTATGAATTTCTAAATGTCAGCTCAAATATATGAGCGAAAAATTAACGCATTATATTCACCAGTGTGCTTCTGATTTTCCCATTTTTTCTTTGAAATTAAATTCATATTTGTAGGATACTGAGCTTGCCAACCGTGCCACAGGATTTAGGCTCTATTTGATAATGCTGTTGGTTTTTCGAGCATTATCAACTTTATTGTACGTATTAGCTTTTATTTCACAGCATTTGTGAGCTTAAAAAGCCAAAATAACCCAATGCCAAATAGAGCCTTAGTTTCTAGCTCTGCAGCCAAGATTTCAAATTTTGAACCCTTACATGATTCATTCCAAAAGAAAAGAAAAAAAGAACCACTAATCCAACGTCTACTCTAAATTTAAAATCCTTTATTTAATATCCTTTCTGTTTGTAAAATAAAATTTCAGTGTAGTATGGGACAGTGAAGACGCGTTAGTTGAGGACTTCGGGATATTTCATGATTATATTTACGTAGCTTATCCAAATATCCGATGCCTGAGTTCAAACTTCTAATCTTTTTTATTCTTAACCAGTGTCTTTCACAATACTTGATAGTGAATAATTTCAGTTGATGAACGAAAATAAAACATGTGTAAAGATATTGACATTTTCAATCGGAGGGAAGCTTGATATTTAGTGAAGCTGTTACTGGATAAGATGAGCGGATGGAATTGAGAGATTTTAGGAATCAAAAAGATCATAGAAAAATCGCGTTAAAAGGACGGTTTAAAGTTGGTAAAATCCAATTATAGAGTTTGAGATTTAAAATTCTCATATCTTGCAAAATCTCATAAGTTAGTTGAGTGATTCAATCATTTCAATCTTTTTTTTTTTTGACCTTTTTCCTGAACATATAAAAAGGAGAATGCGCTCTTTCACTGGCCGTACCCTCGATCACTAGGTCCTTGATTGCTAAGCAAGCCTAGTGAGTTAAAATCAAATCATCTGTTTTGGATCACTGTTTGTTGTAGATCTCTTTGTTTAGAGTAGCAAAAGAACAACA

>Cg9g005230.1 - Up_Stream_Len 2000

GTAATTGTGGAAAAGTTACTTGTTTTCTTCTCCCCAATAATTATCTCTCATTGTCATTAGATCAGATATAAGCGGATTCGGATCCCAGGAATCTGGATAAATCTCGTTAGTCACAGAGCCAAATTTGGCTTGAAATCAGGCTTCATTTTGAGCTTGTAATAGAGATGTGATCTTCACCCAACACATTATAGTAAATTCCAATTACGTCTAAAAAATCATGGGGACTCGAATCACGTAACTTCTGTTTTAGAATGAAAGCACAAGCATTCTCCTCTAATGTGATCAGTACTTCCTACTTGGAACTTCGATATTTGAATTCAACACGTTAAACTCTCTCTTTTTTTGGCTCATTACAAAAATTAGAAAACATATGATCTTAAAAGAAAGATAATATTATATAATTTTCCTTTAGGGTTAGATTCATTTTGAGGAGGAAAAAACGATTAAAGAGTGGTAAAATTTTTGCCGCCACACATGGATGAATTAAATGGTAGTTTTCTTTTCTTCTTTTTTTTTTCCGCTTAAAAGAAGAAACATTGAACACCTACAAAAATAATAACATGAATGCTTTTTAGTTACTTTTGAAAATATGAGAAACAATTGAAAATTATTGATTCCTATCTAAGAACTAAGGGTGTGTTTAGAATTAAGGTGCTGTAACTATTGTGGAAAAATACTGTAACTATAAAAAAGTTAATATTATGTAGTAAATATAAATTTTAAATAATAATTTTAATAAAATTATTAAAGATATAATATATTTTTCATCATACAAATAAAAAAATTTATATCAATATAACTTTTAAACTATAATAACTAGTATTTATTAAATATTTTAATATTATAATTTTTAAATTACAATCACCGTGTTATGTTGCCATAATATGACTATGGTTTGATATAATCTAAATACAAAATTAGATGTAGTAAAAACCAAGAAAAGAAAAGAAGTAAATACAGATCAAGACAAAAAGCAGAATTTTGTTGTCAGTCAATCACTGATTATAATAACGTAACACAACGACAAGCAATCACGTAACCCGAAACTGATACCGCCAGTCCAGTAACTCCAATTTTAAGCATTCAAAAACAATTAATAAAAATGAGCACCCATAGAGTCGGCACAACTATTGTAACAACTCAAACTAAGAAAAAAAAAAAATTTTTTTTTTCAAACCCGGTGGATGATTAAATGATATTAATGACAATAAAACATACATTTTCAAATCCAACGGATCAGACAGAATCAAATCAATGTGAGTATGGGCCACCATCAACACCTTTGATTTTTCTTGCTACCCGCACATTTTTTTATCGGATCAAATGTGCACGTTTTCTCCCCCCAACTGGCCTTTATCAAGCAGACCACAAATTGCACGTTGTTTGCCCATCGACATTCAACTCGATTACTTGACCAAAAGCTATCACCCACAGGCGAGTGTACAACACATCGCTGCCAGGCCAGCATTTTCAAATTCTACTCATTTCACCTGTTGAACATTTTTTCAACACACAGAATTAATATTAAATTAAGAGAAAATAAAAATAAAAAGAGATTTGTGATTTCACACCTATAGCTGGAAATAATAGGAGCCGTTGGTTGACTAGCCAGCCAGCCAGCCAGCCGACGTTGAAATTTCTCACTTAAATATATGACTGTTTATGTTACGTACAAACAATATCCATTGGAATACAAAATAAAATTAAAAAAAAAAACTCCGCCACGTGTAAATATCCTGATGGTGGGGCCCCACTGTAAACTACATCATCCCATTTTCGTACCTCCTCCATTGCTGAAGTCTCTATATATAATAACACACACACACACACCACTCTCTCATTACCACATACTTCATCTCACAATAATCTCTTCTTCTTCTTCTTCTTGTTCTTCTTCTACTTCTTTTTGTTCTTCTTCTACTTCTTCTTGTTCTTCTTCGTAATTACGCTTAACGAGAGAATTAGAATTGGAATATCGTTTCAGTTTTTGATAATTAAGG

>Cg9g023370.1 - Up_Stream_Len 2000

TCAAAATTAAGTAGGACTCACGGAATGAGATTCTCACACCCCCTTAAAATTTACAAGTAAATACTATAATACTCCTATTTTAAAAAGTAAACACAACATTAGTAGATTTAGGATGGCAAGACCAAGATCAAATTAAGCATAAAAATGGATCGGACTTTAACCAAACTTGGTCCATGTGGATTAGCGTTGGGCTTCTATTTCATACACCGTGTAACGGCCTTTGTCTTCCAAAGGAGCCCATCAAGTTTTTCAATAGGTTTTCATCTTGTTAGTGTTAATCGGTTGACATTACCCAATCCATTGGCAAGCGTTAAAGATTCTTCTCCCACCATAATAGAATTTGTTCGAGTCTCTATGAAAGACGATAAATGCCCATGATTACTGAGGATAATTGACCCTTCTAATTTACTCTTTAAAGTAAAATAAATTATTGTAATTTAAGGATTAAATTGAAGCAGAATAACTATAAAGTAGCCCATAATTAGTAACCGCATCTAAAATTGGAAGATCTTTTATAGAAGATATTCGACAGTGCTAAAAAATTGTCAAAAATATTATACCATCTTAAGGAGGAAGTTGATAACAGAATGAGAAAATAGGCCGACATAGAATTCAATGGATTGAGATGAAAATGGAAAATTGGTGAAATTGGAGGATAAGAAGACCACCTAAGTTAATTCTTTTCTCATTACTAAAAAACAATTCCAATTTAATGCAATGAGATAAAATCTTGTGAGGAAACATAAGGTATTCTCCTTCAATAATAATGAAGATTGTGATAAAATGTTAATTAATTTAAAGTTGGTAAATTTTCAAGATTTTCTCTCTTGTTTAATTCGTCCATTCATTCTAAAAAGGAAAGCACCCTTCCCGCTTGATTTAGTAACAATTAATAATAACAAAATCAATACCAACAAAGTATTGGCTCCACTGGCAATTGAGTCTCCTTAAGTGGCTTGACTGGGTTTGCTCCCCAGTTCGAGTCGCAGGGAAGTCGCCTTTGTTGGGAGAACTTGTGCCTCCCGGTTCGAGCGAGGACTTCTAGTTTAGGTTGTGGTACGAGCTTAAAGGTGTCTCCCACAGTTGGAGCCCTTCCCAGGATACTTCGTGGATAAGACCAAAAAAAATTAATAACAAAATCAATTCTTCAAAAGTTTTGAAAAATTTCACTTGATCCCTCATCTTTCCAACGATATTATAGAATCAAAATATCTATATATTTTTAACACAATCTCAATGTGTTTCTTGATATGTATCCTTAAAACCCCCAGAGCTTCCACTATATAATTTTCTAAAAATAATTCTGCTACGTAACAAATTAATATAAAAAAAATTAACAACTTCATGATCACTTTTTTTTGTGGGTTTCTTTTCGTATTTTCAGTCCATTATTTGCTCAAAAAGAAAATTTATATATGCTTCTGGGTTGTGATTGATAATTTCCATTTTTAAATAAAATAGATTGTTTTAAGGGCATAATAAGCAGCACTAGGGCAGAAGAAAGAGGACATCTCACGTCTCTAACAAACAAGGGACCTTTCTGCCTAACTCATAAAGTCAGTCAAATAAAGTAAAATTCAGAGAAACAAATAGGAGCTTTTTGTAAATAAAATTTCCCGTTTCTTTCAACGCTCTAACAAGAAAGTATAGCGCTGGTCCCTACTCTTTGGGGATTTTGTGATAATATAATATTTACTGTCTGACAGCGAAGTTCTGAACCTCTGGTCATCTTCCATGTTCATTTGTTTGTTTCAATCACGTCACTTGCACTCACACTAATAAGTCCAAAGCCAGTGTGGTTTTTTATTTTTAGTAGTGATACCGAAAATCTTCACTAGTCAACAGAGGCTGCTTCCTGCTTGTGTTGTGTTGTTTGAAGTGCTATAGCTTTTTCTACAACTTTTGAACTTTCTTTCTCTCTTTCATAAACTGTATCTCCTTCTTTGTGACTGTGTGTTGTGAGCTTCGGTCAAGTGTTTTAGGAGCGGTAAAGCAAAGCC

>Cg9g005200.1 + Up_Stream_Len 2000

GTAATAAGGACCTAGATTCTTAGATTGAATCATTGATAATGATGTTGCAGTACTCGTTCACTTTATAAACAAAGAAAGTATGTCTCCACTTTCAAATAAGATGCTATGTGCCTGCACCTACTAAATTAAACATTTTGAAACTTATCAGCACCTTATGTTTGCTTGATGAACTGTAAGATAAGTCGTTATATTTTCAGCTTTTTGTTTTCGACTAACCTCGATTTTCTGATGGCAGAGCTCGGCGTCGTGATAAAAGTTTGCTCACAAGGAATACAGAAGCGGAGCATCCCCTTTTGAATGGTCTTTTGGTTGAAGATCATAACTGATATGTAAGATCTACACTGAAGACCAATTCCTTTGGCAGTCTGCTCCCACAAATTTTTTCTGCTCAAACCAGATTTTTTGTTTTTCTTATAGGATGAATTGTTATTCATTGATTACTGTCCCCGTTGCATACCGGGGATGGACCACACCATGAAAATATTGTAACATAAATCTAATAGTAAATTGAAATATGTAGTAATTATTTTTTCTTTTTAATTCTTTATTGTACTGGAATGGATCATGGTTCCTGGATATGGCTATAGTTGCCAAAACATAATATGAGTAATGCAAAATTTTTGTCTCAGACGAAAATGTTCAAAATTTTAATTTGAATTGTTTGTATCAAGAAAGTAATTGTAGTAGCATTCGCTCTCTGTTCAACGTATGAAGCACGAAACGTCAGATGCCATATAAAGTTCCAAAGATGGGGTAGCTGGAAGCGATAGTACAAGGCCGAATCACAGTGGTTTGGCGGTGGCATATTGTCAGTTAATTGAGGATCATCACAGAGGATGAATGGACGATGGTCAAGGTAATAGTGGAGGTTTTTGAGTCTCTGTTCACTATTTAGTGAAGTCAAAAGAAGCTTAATGAAAGTGAATTTTTAATTTTATTAATCCTTGAAAATCGTCAATCTCTGAAAAACTCAAGTTGTAGAAAACCCCGGAAGTAACTGCCAGATTTGATAGCCCTCGAAGGCAAATTTACTTGGGAAACATTTTTCCATAGCCTGTCGTCATCAGTATGACAAAACACGCTAGCGCCTATACCGGCTGTCAACATTATTTGAAACTTTTGTTCGCTGGATAGCAGAAAACATACCCGTATATTCTCGATCTATGCTACGTAGATCTTCAAATAAAAGAAACGAAAAACATACAATGACGTATAAAGGGTCTTACAGGCTATCCGTTAGCGCGGGTTGGAGCATTCTATTCTTCTGGTTACTATAATTGCTAAGCTACTCCTCTTTTACCAGATAGAAAAAGGGCCTTCACTGTTCTTCGCGGGAATCTTTCGTCTCTTCGTGGGAATTCAATGCCATGTGATTCAAGAAATAAGAGTTCCACATCTTACTAAGTTACTATTGTTAAATTCTTTTGTAACCAATTCATACGTAATTAATAATATAAGAAATATTGATACCGAGAGAGAGGAATATTTCAGTTGGTATCGACGTTCATACTTTTAAATTTGGTCTTAGTAAGATGGTAATAAGTTTTGAGTGTGGCTACGTATTTAAGAAGTCCTTCCTACAGATTAAGAAGCTGAAAATATTTGAGATCGACACGACGACGCTGTATGTGTTTATCCACGAAATAACACGCACATGAAATTGAATTGACTAAGTTTACACAAGGAAGATGGGGTCAAAATTTAGGCAGTGACCTACGGTTGCGGACAACCCAGTTATCATTCCATATTTTAAGGGCATTACGGATTTACAGTTCACGAAGCACAACTTATTGTCTCTGCGTCTGCTCTTTATAAATAATCAACAAAACCCAGAGGTTAAAATTTACAGCCTTTCCTGGATCCCCTTACTTTGGGTTCATATTTTCCGTCCGCCTGCTGATCTCCCCATTGCATGTAAATATATAACGCATATAGTTTCTTCATTATCATTAGGATTCATTTGGTACTAATAAATATATATTTACCTTTGGGGAGTCAAA

>Cg9g005210.1 + Up_Stream_Len1041

TATCAAATTTCTTTCATTAATGGTTCTCAACTTTCTATTTACTCACTTTTTCATTGTTTTTTTTACTTTTCTTGTTTCGTTTCTTCATCACCATGCATGTTCGAAATGAATAGACGAAAAAGTGGGACTTTTTTAACACTATGGTATTGGTATATACTGTGAATACATTGATCATTACATATTCTTTACACTCCCAAGCGTGCTGGGAGAAAATTGATGAGTCTCTATGTATACATATGATTCATAATCTAATATTAACTAAGGAAATCAAATTATTGAATTTGAAGAACATTTTACATAATTATGAATTCACTTAACAAACTTTGGATACTATGAAATCTTGTACAACTTCTAACCTTACAAACAAATTGATGCGACAGTGTTGTGTCTTTCATAACTTCATCAGTTCTACAACACGACTGTTCTATTGCTTTACTCACATCTCCACAGCTCATTTCCCTCAGAGCATGTGTCGACTTCCTATAAATTCAACCGAATTTTTCCCTCCAGCATCTTTGAAACCTTTCGTTTTCTTTAAAATCTTCCCAAATTTTTTTCTTGACATAATCTCCTAATAATTAGACCATGCTAAATTATATTGGTAAGTACAAGGGCATCCTCTTTGTTATTTCCTCTTTTTTTCCCCTAGTATAAGGGAAAATTTTGTCCTTAAATGGCAACGATTCTGGCAATTTCCAAATAAAGCAGTTTAAATTCCTTCAAAAGTATATATATATATATAGAGAGAGAGAGAGAGAGAGAGAGAGACGAATTCCAAGGTACCAATGTTAAATGTGATTCTTATCTTCTATCCAGCCAACGTGTCATCCCAAGTAAGAAATTAAAATCCCATAATATCCCATATTTATGGGAATCATTAAGTTTAATTTCCAGAATTCCAGAATAAAGTAGCAATAAATTCAATGAAGAATTTAACGCTAAAGCAGTGAACTTATTACATTTTCTCTTGTTCAATTTCTACTTTTTTTTCTCTTTTTGCTCTTGGAAAGTCATATTATCATATACAATTTGATCGCAAAG

>Cg9g023330.1 - Up_Stream_Len 2000

ATATTATAGTTTAATCCTTTCTAATAACATTTTGCAGTCTTTGAGTCTTGACTAAGCCTTTCTGTATGGTTAGTAAGAGCCGATTTAAATTCTCAAACGAAACTTAAAAATTTAATTGTGTATATTTATGCTAAGGTGGAGTGACATCAATGGAGCCATTTTTGAAGAAATTCTTCCCAGAAGTGTACAGAAAAATGAAAGAAGACACCAACATTAGCAATTACTGCAAGTTTGACAGCCAACTATTAACCACCTTCACGTCCTCTCTATACATTGCTGGCCTTATTGCCTCCTTATTTGCCTCCTCAGTCACCAGAGCCTTCGGCCGCAAGGCATCAATTCTTGTCGGCGGCACAGCTTTTCTTGCTGGTTCAGCCCTCGGAGGCGCTGCATTTAACATTTACATGCTTATATTTGGGCGGGTGTTGCTTGGCGTTGGCATTGGTTTTGCAAACCAAGTAAGTGTTTGGTTATTTTATTTTTTCCAAGCATTTGATTTGCTCAACAATTTTCAATTGGTTTTAATTTGCTGGTTGCAGTCAGTGCCACTGTATCTCTCAGAAATGGCACCACCAAAAAACAGAGGAGCGTTCAACATTGGCTTCCAAGTATGTGTTGCCATTGGTGTCCTATCCGCTAATCTTCTCAATTACGGCACCCAAAAGATCAAGGGTGGCTGGGGCTGGAGAATCTCCCTGGCAATGGCTGCTGCTCCTGCATCAATATTAACAATAGGTGCACTTTTCCTGCCAGAAACACCCAACAGCATAATCCAGCGCAGCAATGACCACCAAAAGGCTGAAAGGATGCTGCAGCGTGTGCGTGGCACAGCTGATGTTCAAGCAGAACTCGATGATCTCATCAGAGCAGGCTCCATTTCAAAAACCATTAACCATCCATTTAAGAAAATCATTGAAAGAAAAGACAGGCCTCAGTTGGTAATGGCAATACTGATACCATTTTTCCAACAGGTGACAGGAATCAATGTCATTTCATTCTATGCTCCCGTACTCTTTAGGACAATCAAACTAAGTGAAAGTACATCACTACTCATGTCCGCAATTGTGACTGGTGGTGTAGGTACAATTTCAACAATCTTATCGATGATTCTAGCCGATAGACTTGGCCGAAAAGTATTGTTTTTAGTTGGGGGGATACAGATGCTTGTGTCACAAGTAATGATTGGATCAATCATGGCAGCTCAACTTGGTGATCACGGAGGATTCAGCTCAGGCTATGCTTACTTAATTTTGGTTCTAGTTTGTGTATATACTACTGGGTTTGCTTATTCATGGGGGCCATTGGGATGGTTGGTTCCAAGTGAAATTTTTCCACTAGAGATTAGATCAGCTGGGCAGAGCATTACTGTGGCAGTCGGTCTTTTGTTTACTTTCTTGGTTGCTCAAACATTTTTAGCCATGCTTTGCCATTTTAAGGCAGGGATTTTCTTCTTCTTTGGAGGATGGGTGGCATTTATGACGACATTTGTGCACTTCTTTTTACCTGAGACTAAAAATTTACCAATTGAGCAGATGGATAAACTGTGGGTAGAGCATTGGTTTTGGAGGAGAATAGTTGGGGAAGGAGTGGAAGACAGTAAGATACAAGAAGCACTATAATACTTGTAAGGGATCCCAGGAAACAATAAACTCATCACTTTTTCCAAAGGCTGGCTAGCTGCTCTTTCTTCTTGCTCTGATAGTGAAGCCAAATAAAACTCCAAGCTCTGATGTTAATTCTTCAAAATATTTGTAACAGCCCCACATGCTACTATAATAATATATATATATTTTTTTTTATCATAGTGTTCCATGTTGATCATATGGTGATGCTGATATTATTGTCTACGCCTACATAATTCAAGAGAGAGAGAGAGAGATCCTCTTACTTTATTAAGCCTTCACTATCTGCAAAATTTTGAACACACTACTCTGTCTTCTCGTGTCGTCTTTCTCTTGCTGTGAGTGCTGATCAGTTTTGAAGATTAAGCAGCAGCAGAT

>Cg9g023340.1 - Up_Stream_Len 2000

TCTTCTTGAAGCCGTTATTTGCCAATTTCAACCTGCACTCCCACAGACTTCTCAGTTCCCACCAATAATTATTCACTCTTTCAGCCATGATGCATTCTCAAAACGAATAAACAATGGTTTCTCGAGAACACTTGTAAATAAAATCCCTTTTATAAAAATATCTTAATTTTATTAAAATATTATAAAAGAAAATAAAAGAAAATAAATACTTATTTGAATAGCGGGCAGAGGGAGCATTGGTTTTGTGGGGTAGTGGACGACTTGAGTTATTAAACTTAGCTTAATATGGCAAATATTTGCAGAGCAAACATCAGAGAAAAATGTTACCATTTCATTGATGATGCTTGAAAAATGAAACGGTGAATACAAAAGTTGTTTGTATATGCATACAAACAGGGCAAGCCGTAAGATAAGATCGGCTCACTAAATTCAGCCTCTGCTGGCACAAATCCAGCCATCAGTTATCATCTAGGATTCTTTCCTATGCATATGCTGATTGCTGAGGTTGCTCTGTGCAGTTGTTTGGCCATGTCACTGTTGCCACTTCATCTGCTGACTTATTTTGCTTCACAGATACTGCGGCCGGCTTCCTCGGCATGTTAACTGTATTATTACGTGTGCTTCTTGGTTAGTATGTGTTGATTCTTCACAGCAGCCATTATTATCAGGATGAACATGGTATCTTAAGGCATGATATTTATAAAGTAATTATTCAATCAAGAAATCTTTTATTTTAGTAAAGTGAAAAATAAATGAAAAAAAAAAAGAAAGAAAGACAAGGAGTATATACATTCAAAAATTAACTTTATTAAGTGGCATGACTTAGAAATATGTTCGCAGGAAAAAAAAATAGAAAAAATGACTCTATGTATATAGAGTCCTTCTATAGTGCGGGCAGCCACTTTTTTTTTTTTTTATTCATGCGAATATATTTTTAAGACATGCAGTTTAAGAGAGTTAATTTTTAATACTATTCAATCACAGAGTTTTATATTTCATTCAAAAATAAGTTTGTTAAGCTCATAGTTTAAAGCGTATCCGCATTAAGAAAAAATGAAGACGCCCGCATATGAGAATGATTATACACACACACACACACGTGTATATACATTAGTAACAGACTAGTGAATCAAAGTCAAATTTGTCTTTTAGAAGTCCTTTCTTAATTTTTTTTTAAAAATTTTTAACTTGGTAGTAAAAAATTATTAGACTTCCAAGTTTTAATATTTATGCTATGAATTCTGTTATTTTGACTATTAATGGCAAATTTTGGGGCTCTAAGCAAAATCAAACAGACTGCACACCAACTTTGACTTGTCAATTCATCATAGTTGATTATTTTGTAAGAGATTCTGCGCTTAAAAATCCGGTGATTCTACTTGACTTCGAAAGCTCAATAACTAGATATTTGGTAACATTCGCTATTTTAATCTTTTGATAATTAGCAAGTGACTCTTCTTCCAGAGCAGTACACAACACCTCAATTGATGGCTATAAGCGCAAAGCAATGATATTTCTGGGTTACTGATCCAATATATTATACTTACATGGATTTCTTGAAGCACAACATCTTAAATTTTCCAATGAGGTACTTAGCAGCTGCTATTTTTATCTTGACTCCATATGCTTTCACAATGATTAGTTCTTCAATATTCAACAACCCCACGCATAGATTGCCACGTGTTTTTATTTTCTTAATTTGTTTATTTATTTATCAAGGCACGTACATTAGTGAAATTTGAACACGGACCTTGGCCTCAAAGCACACGCTGCCATGTTGATCATAACTCCAAAGCCATGCATGACTAACGGTGCTGATATTAGCGTATACGGCTACTAGTCAAGAAAGACTGCAGGTACTTTTAAGCCTTCGCCTTCTGCAATTAATCTTCAACACACACACACACACACCGCTGTCTCTTTTCTCTTCTTTATATAAGTTGCTATGATCAGTACTCCTGATCAGTTTTGAAGATTCAGCTTAGCTATCCGCAGTCGAT

>Cg9g023350.2 - Up_Stream_Len 2000

AACATTAGGTGCACTTTTCATGCCAGAAACACCAAACAGCATAATCCAGCGCAGCAATGACCACCAAGAGGCTAAAAGGATGCTGCAGCGTGTGCGCGGCACAGCTGATGTTGAAGCTGAACTCAATGATCTTATCAGAGCAAGCTCCATCTCAAAACTATCAACCACCCATTTAACAAAATCATACAAAGGAAATACAGGCCTCAGCTAGTAATGGCGATACTGATACCATTTTTCCAACAAGTGACAGGGATCAATATCATTGGGTTATACGCTCCTGTACTACTCAGGACGCTTAAACTAGGTGAAAGTACATCACTACTCTTGTCCGCAGTCGTGACTGGTGGCATAGGTACAGTTTTAACAATCACATCAATGATTCTAGTCGATAAACTTGGCAGGAAAATATTGTTTTTAGTTGGGGGTATACAGATACTTGTTTCACAAGTAATTATTGGTTCAATCATGGCTGCTGAGCTCGGTGATCACGGGGGATTGAGCCAAGGCTATGCTTACTTAATTTTAGTCCTGGTATGTGTATATTCAGCCGGGTTTTCATATTCATGGGGGCCATTGGCATGGTTGGTTCCGAGCGAAATTTTTCCATTGGAGATTAGATCAGCAGGGCAAAGCATTACCGTTGCAGTTACCCTTGTGTTTATTTTCTTCAGTGCGCAAACACTTTTAGCGATGCTTTGCCATTTTAAGGCAGGGATTTTCTTCTTCTTTGGAGGATGGGTAATGGTGATGACTACATTTATGCACTTGTTTTTGCCAGAGACTAAGAATGTGCCGATTGAGCAGATGGATAAAATCTGGAGACAACATTGGTTTTGGAAGAAATATGTGGGTGACGTGGATGAAGAGGGTAAGATGTAAAAGGCCTAATTTGTCCCCAAGGGTGTAATCGGGTTGGTAACAGCATAAAACTTGTAGTTACTGATCATCCAAATTCGATTATGGGTGTAGTAAGGGGGGAAAATAAAATAAAAATTATTTAGTCTTTACCCACTCCTTACTTTATTTAAAAGAAAAAAAAAGAAAAAAGACTCGATTTCTACTTAATTTTGCCTGTAAGTTTCAAACTTTCAATATTTGTGCTCTGCATTTTATTTGACTTATTAATTCGCTGTAATTATTTTGTCGGAGATTCTTTTATGCTGTGTTTAAATATATGCACTAAGCCCATCAAAATCCTTTTCTTGAGAAAAGCTGCCAAGTCCATCAAAATCTATAGACATGCCATGGTTAAACGCATTGAATAACAGCCCCCTTGAAGTAAATAACACCTGAAGCAAATAATCACTTTCCTTTTAACAAAAAAATATTTTAATAACAAAAAATTTATTTTTTTGAATGGCGTTTTGAGGAACATTGATTTATTTAAAAGAATTTCGTGGGCAAAGTGGATGGGCATGGCAACATGGAAGAGAAATGATATTCATTTTATCGAAATGAGGATTAAGTTAAAAGATTAAAATAAATACATATATATTTAAATATTTTATTTTATTTTTCAACAAAGTATATATATACACACACACTTACTTTTCATTTGATCTTTATTTTAGTGAAATGAAGATTTTTCTCCCGGACCTACATGCATTTAGATCAACAAAGGTGTTAGAAAAAAGGTCCTCTTTGACTTGATGACTAAACGTTTGGTAGGACTCAGCAGTTTTACTTTGATAATTGGCCGCTGACTCTTTATCAAAGCAGTTGTGAATTACACGTAGCACCTCGCATTAATTAGTGGTCAACAGTCAACAACCACAAACCCCGCTTACGGCTACCTTGATTACAAGGTCATAATGCTGCTAGAAAAAGGAGAAATATTTTAAATTTTTTATCCTAAATTTTATCTTAAATAATATACTATTTATTGATTGATTAACAGTTTTTTACAAGATTCAAATAAATTCTAACTTTTCTCACTCTCCTTTCTCTCTTGCTGTGACCTGTGAGTGCTGATTAGTTTTGAAGATAAAGCAGCAGCCAAT
